# A systematic review of the effects of shared decision-making in the South Korean healthcare system

**DOI:** 10.3389/fpubh.2025.1667803

**Published:** 2026-01-05

**Authors:** Hyunok Yun, Kyung-Sook Woo, Do-young Lee, Sang-Ho Yoo

**Affiliations:** 1 Department of Public Health Sciences, College of Medicine, Hanyang University, Seoul, Republic of Korea; 2 Institute for Health and Society, Hanyang University, Seoul, Republic of Korea; 3 Department of Medical Humanities and Ethics, College of Medicine, Hanyang University, Seoul, Republic of Korea

**Keywords:** shared decision-making, patient-centered care, patient outcomes, systematic review, South Korean healthcare system

## Abstract

**Introduction:**

Shared decision-making (SDM) is a collaborative process that improves patient-centered care and has been widely adopted across healthcare systems internationally. Despite increasing attention, SDM remains underutilized in South Korea, and systematic evidence on its implementation and effectiveness is limited. This study systematically reviewed SDM programs implemented in South Korea, assessed their effectiveness, and aimed to inform the development of context-specific models for broader integration into healthcare practice.

**Methods:**

This study employed the ECLIPSE (Expectations, Client groups, Location, Impact, Professionals, Services) framework to refine the research questions and conducted a systematic search across seven international and domestic databases, as well as Google Scholar for studies published until July 2024. Eligible studies included quantitative designs that assessed the outcomes of SDM interventions. Study quality was assessed using the QualSyst tool, and a narrative synthesis was conducted due to the heterogeneity in study designs and outcome measures.

**Results:**

Of the 14 included studies 13 addressed information provision, 8 involved patient participation, 7 incorporated decision-making processes, and only 3 integrated all core SDM components. Outcomes were reported across three domains: Affective–cognitive (e.g., satisfaction, decisional conflict, knowledge), behavioral (e.g., intention to act), and clinical (e.g., quality of life). SDM was associated with increased patient satisfaction (in 10 out of 14 studies), reduced decisional conflict (in 8 studies), and improved patient knowledge (in 9 studies). However, variations in effectiveness were observed depending on patient characteristics, healthcare settings, and the design and delivery of interventions.

**Conclusion:**

This review highlights the potential of SDM to enhance patient-centered care in South Korea. However, inconsistencies in intervention components and evaluation methodologies limit the generalizability of the findings. Given the unique features of the Korean healthcare system—such as very short consultation times, limited reimbursement for counseling, and provider–patient information asymmetry—future research should focus on developing SDM models tailored to these constraints and cultural contexts, supported by appropriate evaluation tools and policy measures.

**Systematic review registration:**

https://www.crd.york.ac.uk/PROSPERO (CRD42024582894).

## 1 Introduction

Shared decision-making (SDM) is a process in which patients and healthcare providers discuss available options and collaboratively make medical decisions based on mutual agreement (1). Introduced by Veatch to emphasize patient rights in healthcare (2), SDM has been implemented primarily in clinical practice in Western countries since the 1990s. It is widely recognized as a core component of patient-centered care (3) and a method for enhancing the quality of decision-making through improved communication (1). Many studies have shown that patient engagement through SDM improves medication adherence, physical health, quality of life (QoL), and satisfaction with services, while also reducing hospital and emergency department admissions (4–8). SDM has further been shown to enhance patients’ knowledge of treatment options and reduce conflicts related to patient preferences and value-based decision-making (9).

As evidence supporting the ethical, clinical, and practical value of SDM continues to accumulate, several countries—such as Germany, the United Kingdom, and the Netherlands—have institutionalized SDM through national policies and healthcare regulations (10–12). Additionally, countries including the United States (13–15), Japan (16, 17), China (18, 19), Saudi Arabia (20, 21), and Ethiopia (22, 23) are actively conducting national-level literature reviews to evaluate their implementation, key outcomes, and contextual applicability as part of efforts to expand and formalize SDM practices.

A review of SDM implementation and outcomes within individual countries highlights several key insights. Evidence indicates that SDM and PtDAs can reduce or sustain healthcare costs and utilization without impairing preventive care (14). SDM also supports the transition to value-based care and improves patient engagement, satisfaction, and health outcomes across diverse populations (15). In cancer screening, SDM combined with decision aids enhances patient knowledge, promotes informed choices, and reduces decisional conflict, enabling more personalized participation (16). Additional reviews identify factors affecting SDM adoption, such as patient, provider, and health system variables, and recommend efforts to enhance health literacy and provide targeted support and training for effective SDM use (20–23).

In South Korea, the adoption of SDM remains limited despite the increasing recognition of its importance. Although the Life-Sustaining Treatment Decisions Act was enacted in 2018, allowing patients or their legal representatives to determine whether to discontinue or withhold life-sustaining treatments (24), there are no laws or systems explicitly incorporating the concept of SDM. This policy gap continues to hinder stable integration and widespread implementation in clinical practice. Furthermore, several substantial challenges impede effective communication between healthcare providers and patients. Chief among these issues are the brief consultation durations and inadequate remuneration for medical services within the Korean healthcare system. The current reimbursement structure does not sufficiently compensate for patient counseling, resulting in the normalization of so-called “3-min consultations.” Such time constraints place a significant burden on healthcare providers and serve as a barrier to implementing SDM. Additionally, the asymmetry of information and knowledge—where providers possess more expertise than patients—creates a power imbalance that further obstructs patient engagement in decision-making. Given these circumstances, the urgent need to adopt SDM is evident, as it has the potential to significantly enhance patient participation and improve communication dynamics within the Korean healthcare setting (25).

The increasing awareness of the importance of patient participation in the treatment process has led to progress in implementing SDM through collaboration between patients and healthcare providers in specific clinical areas of Korea. Since the mid-2010s, SDM has been piloted and gradually expanded across various clinical settings, particularly in oncology (26, 27), with several experimental studies investigating its effects on patient outcomes (28–30). More recently, in 2023, the South Korean government launched a four-year national research initiative titled “Shared Decision-Making between Patients and Physicians,” funded by the Ministry of Health and Welfare, reflecting growing institutional recognition of the importance of SDM in clinical practice (31).

Despite these developments, no systematic review has yet been conducted on South Korean studies that provide an overview of SDM implementation programs and assess their effectiveness. Considering international policy advancements and the expanding body of SDM research, a comprehensive evaluation of SDM implementation and impact within the South Korean healthcare system is urgently needed. Such an assessment will provide critical insights into the current status, benefits, limitations, and contextual challenges of SDM in South Korea. A scientifically grounded synthesis of the evidence will support informed policy decisions, including the development of a South Korea-specific SDM model and the design of pilot programs for potential integration into national health insurance coverage. In evaluating the impact of SDM interventions, patient outcomes extend beyond clinical health indicators to encompass cognitive, affective, and behavioral aspects of the patient experience. Therefore, this review adopted the conceptual classification of patient outcomes proposed by Shay and Lafata (32), which organizes outcomes into affective–cognitive, behavioral, and health-related domains.

This study systematically reviewed SDM programs implemented in South Korean healthcare, summarized their core features and outcomes, and assessed their impact across various healthcare settings, patient populations, and healthcare professionals.

## 2 Methods

### 2.1 Protocol and registration

The protocol for this systematic review was developed in advance and registered with the International Prospective Register of Systematic Reviews (CRD42024582894). The review was conducted and reported in accordance with the Preferred Reporting Items for Systematic Reviews and Meta-Analyses (PRISMA) guidelines (33).

### 2.2 Eligibility criteria

This study employed the ECLIPSE model (Expectations, Client groups, Location, Impact, Professionals, Services), a useful framework for evaluating policy or service performance, to define eligibility criteria and structure the research questions (34). Expectations (E): Examined the potential impact and effectiveness of implementing and disseminating SDM systems. Client group (C): Literature on adults aged ≥18 years who are capable of making healthcare decisions, excluding cases where surrogates made decisions for patients aged <18 years or for those lacking decision-making capacity. Location (L): Focused on South Koreans receiving services from South Korean healthcare organizations, excluding foreigners in South Korea and South Koreans abroad. Impact (I): Included studies examining patient-related outcomes post-SDM intervention, focusing on cognitive, behavioral, and health outcomes, as well as any unintended consequences, while excluding those that did not quantitatively assess outcomes. Professionals (P): These included physicians or nurses. Service (S): Studies where medical decisions were made through SDM. These studies should assess the core aspects of patient involvement in the decision-making process and address one or more of the following items: in-depth counseling (education and information sharing), treatment option explanation, patient involvement in the decision-making process, and the usages of decision-aid tools. We excluded studies on SDM validation, reliability, evaluation tools, and patient decision-aid (PDA) tools, as well as those addressing life-sustaining or end-of-life decisions, including advance directives.

The research questions derived from this framework were as follows:

- What SDM models have been implemented in South Korea’s healthcare system?
- What are the primary outcomes of SDM within South Korea’s healthcare system?
- What are the differences in the effects of SDM based on patient, disease, and medical provider factors in South Korea’s healthcare system?

### 2.3 Search strategy and study selection

We systematically searched both international and domestic databases for quantitative studies published through July 2024. The international databases included MEDLINE (via PubMed), Embase, the Cochrane Library, and Web of Science. The domestic Korean databases included ScienceON, RISS, and KMBASE. The primary search term was “shared decision-making.” Search terms that could identify patient-centered care and patient participation in medical decision-making were added to the search (Appendix B: Supplementary Tables 1–7). Medical Subject Heading terms were reviewed and incorporated to enhance sensitivity and specificity. Similar search strategies were applied across all databases. The search was restricted to studies conducted in South Korea or involving South Korean participants by adding “Korea” as a Boolean operator. Filters were applied to limit the results to human studies and articles in either English or Korean. To minimize search result bias, we used Google Scholar as a supplementary resource for identifying potentially relevant gray literature. To ensure sufficient coverage, advanced search techniques were employed, including the use of targeted title terms and a minimum of 200–300 records were screened (35, 36). Finally, the reference lists of the selected studies were reviewed.

The inclusion and exclusion criteria were determined based on the ECLIPSE model-derived research questions (Table 1, Appendix A), and two review authors (H and DY) independently assessed study eligibility. Initial screening was conducted based on the title and abstract, followed by a full-text review, with the reasons for exclusion documented. Any disagreements were resolved through discussion and consultation with the other authors (KS and SH) until a consensus was reached.

**Table 1:** Eligibility criteria.

Inclusion criteria	Exclusion criteria
Studies published up to July 2024 on the effectiveness of implementing or disseminating SDM systems that explicitly use terms such as *“shared decision-making”* or *“participatory decision-making,”* or describe interventions designed to facilitate SDM between clinicians and patients. Adults (aged ≥18 years) with decision-making capacity. South Korean participants receiving healthcare services in South Korean institutions. Outcomes related to cognitive, behavioral, clinical effects, and patient experiences. Study designs: RCTs, non-randomized controlled trials (e.g., pre–post, with or without controls), and observational studies (cohort, case–control, cross-sectional).	Surrogate decision-making involving participants aged <18 years or lacking decision-making capacity. Foreigners in South Korea or South Koreans living abroad. Life-sustaining or end-of-life decisions (e.g., advance directives). Validation or reliability studies of SDM/PDA tools or assessment instruments. Studies without predefined outcomes or lacking quantitative outcome assessments. Non-Korean or non-English publications.

Literature collection and duplicate checking were performed using EndNote 21.

### 2.4 Data extraction and synthesis

Three stages were implemented to ensure accurate information extraction. First, a researcher with extensive experience in systematic reviews randomly selected three articles for preliminary analysis and established uniform standards for data collection. Second, the reviewers were trained to achieve uniform standards for data extraction and coding before the extraction of information. Third, the remaining studies were independently coded by the reviewers using a standardized information extraction form. Disagreements regarding data extraction were resolved through discussions among the researchers. For each study, data were extracted from the bibliographic details, study design, participant characteristics, healthcare processes performed, components and measurements of each SDM implementation, outcomes, analyses, and findings. Three reviewers (H, KS, and DY) independently extracted data, followed by multiple cross-reviews to ensure accuracy. Any coding errors were addressed by closely examining the articles until discrepancies were resolved through consensus. We analyzed outcomes using narrative synthesis, a technique for exploring relationships between studies after the preliminary synthesis of findings (37, 38). This approach was selected to avoid potentially biased or misleading pooled estimates that could arise from the substantial heterogeneity in study designs, populations, and outcome measurement criteria. Meta-analysis was deemed inappropriate given these conditions, which are often encountered when the number of included studies is limited or when substantial heterogeneity exists (39, 40). We systematically categorized key findings by domain within and across studies, assessed the direction of effect for each (positive, negative, or null), and used this information to develop structured summaries and descriptive statistics. The synthesis results are presented as tabular summaries of key findings, complemented by narratives that describe observed patterns (41).

### 2.5 Conceptual framework and classification

The conceptual framework for this systematic review is adapted from models proposed by Shay and Lafata (32), which are based on communication models between clinicians and patients developed by Kreps et al. (42). According to this model, patient outcomes should be categorized based on their impact on the individual across three domains: affective-cognitive, behavioral, and physiological. Affective-cognitive outcomes include knowledge, attitudinal changes, and emotional or affective effects. Behavioral outcomes encompass adherence to recommended treatments and the adoption of healthy behaviors. Physiological outcomes (health outcomes) include measures of quality of life (QoL), self-rated health, and other objective health indicators (32).

Using this framework, we reclassified patient outcomes reported in the included studies into three categories: affective–cognitive outcomes, which encompass emotional effects, conflict, and knowledge/attitude; behavioral outcomes, which include problem-solving ability, willingness to pay, and intention to undergo screening; and health outcomes, which involve QoL, self-rated health, and objective health measures.

### 2.6 Quality assessment

Here, quality was assessed using the QualSyst tool, a checklist for evaluating quantitative studies developed by the Alberta Heritage Foundation for Medical Research in 2004. It has been widely used in recent health-related systematic reviews, and its validity and utility are well established (43, 44). The QualSyst evaluation criteria included clarity of the research purpose and question, rationale for the research design, definition and source of participants and variables, participants characteristics, sample size, appropriateness of the analysis method, control of confounders, and accurate reporting of results and conclusions.

Some items, including the clarity of the research problem and context, may be interpreted differently owing to researchers’ subjective perspectives. To address this, three researchers (H, KS, and DY) independently evaluated the items, and any disagreements were resolved through discussion until a consensus was reached. Each item was scored based on the extent of satisfaction: 2, 1, and 0 points if fully satisfied, partially satisfied, and undescribed, respectively. The total score was calculated using a maximum of 22 or 28 points, depending on the study design, and the final score was derived by dividing the obtained score by the maximum possible score (45).

## 3 Results

### 3.1 Search results

**Figure 1:**
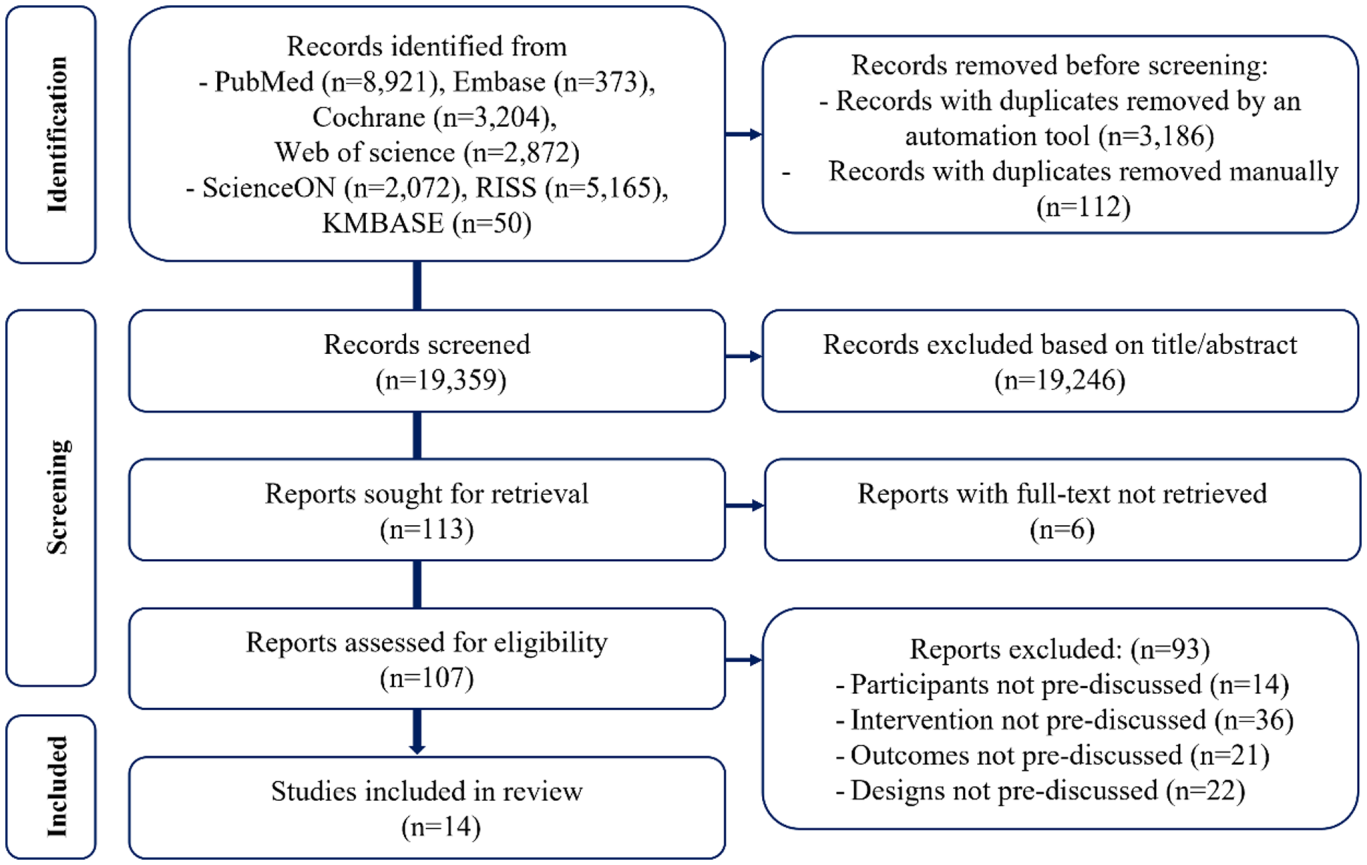
PRISMA flow chart for inclusion in the systematic review.

The initial search yielded 22,657 records. After removing duplicates, the titles and abstracts of 19,359 studies were screened, and 107 full-text articles were assessed for eligibility. Ultimately, 14 studies met the inclusion criteria and were included in the review (26, 28–30, 46–55). No additional studies were identified through gray literature searches. Reasons for excluding 93 full-text articles are provided in Supplementary Table 8 (Appendix C). The study selection process is summarized in Figure 1.

### 3.2 Characteristics of included studies

Among the 14 included studies, one was a randomized controlled trial, two were quasi-experimental, three were one group pre-post studies, and eight were cross-sectional. Most studies were conducted in secondary or tertiary hospitals, with only one study being conducted in a community setting. While three studies included only male participants, the others included both sexes. Each study included a diverse patient population, with three studies focusing on patients with cancer. Regarding SDM components, 13 studies included “information provision,” eight studies included “patient participation,” and seven studies involved “decision-making” processes, with overlaps across studies. However, only three studies incorporated all core SDM components—information, participation, communication, and decision-making. Two studies used the SDM-Q-9 to measure SDM, eight adapted existing instruments to assess types of SDM, and four did not (Table 2).

**Table 2:** Characteristics of included studies.

Study, year	Design	Healthcare setting	Participants	Shared decision-making		ROB (score/1.0)
			Condition, size (male %), age	Framework	Instruments	
Suh and Lee (28)	Cross-sectional	Secondary hospital	All disease, 288 (64.6%), age (years): 20–44 (55.2%), 45+ (46.8%)	Information provision	SDM-8 item scale	1.0/low
				Patient participation		
				Decision-making		
Min and Suh (46)	Cross-sectional	Military hospitals	Injury/all disease, 514 (100%), age (years): 20–30	Information provision	SDM-8 item scale	0.77/low
				Patient participation		
Nam et al. (47)	One group before and after	Tertiary hospital	Carpal tunnel syndrome, 85 (4.7%), age (years): 53.0 (range 31–76)	Information provision	Control preferences scale	0.82/low
				Patient participation		
Hwang et al. (29)	Cross-sectional	Anesthesiology department at a tertiary hospital	All patients scheduled for elective surgery, 257 (54.1%), age (years): 20s (22.5%), 30s (18.7%), 40s (30.2%), 50s (23.3%), 60 + (6.2%)	Information provision		0.68/high
				Patient participation		
				Decision-making		
Yoon et al. (48)	Cross-sectional	Cancer centers of university hospital	Patients with cancer aged 65 years or older, 109 (66.1%), age (years): 64–74 (65%), 75–84 (35%)	Decision-making	SDM-Q-9	1.0/low
				Information provision		
				Communication		
Sim et al. (50)	Cross-sectional	Cancer centers of a tertiary hospital	Patients with cancer, 625 (41.1%), age (years): 50, 179 (28.6%); 50+, 441 (70.6%)	Information provision	EORTC QLQ-INFO26	0.91/low
An et al. (49)	Quasi-experiment	Mental hospital	Schizophrenia, 60 (100%) (SDM training group 29 vs. control group 31), age (years): 19+	Information provision		0.86/low
				Patient participation		
				Decision-making		
				Communication		
Gong et al. (26)	RCT	Tertiary hospital	Carpal tunnel syndrome, 66 (21.2%) (decision-aid group 32 vs. control group 34), age (years): case 53 ± 10, control 52 ± 9	Information provision		0.71/high
Koo and Lee (51)	Cross-sectional	Cancer centers of university hospital	Preoperative cancer, 110 (34.5%), age (years): 59.7 ± 7.8	Decision-making	SDM-Q-9	0.95/low
Kim (52)	Cross-sectional	Orthopedic hospital	Unclear, 194 (64.3), age (years): 20+	Information provision	Value co-creation 6 items + Gallan et al.’s (68) 3 items	0.86/low
				Patient participation		
				Decision-making		
				Communication		
Sohn et al. (30)	Quasi-experimental	13 outpatient clinics of tertiary hospital	Serious disease (cancer, stroke, coronary artery, rare and intractable disorder), 416 (42.5%) (consultation group 285 vs. control group 131), age (years): case 39.3 ± 26.1 control 32.1 ± 24.8	Information provision	Patient-centeredness Scale	0.68/high
Kang (53)	One group before and after	Orofacial clinic of a tertiary hospital	Temporomandibular disorder 131 (11.5%), age (years): 37.9 ± 14.7	Information provision	Control preferences scale	0.82/low
				Patient participation		
Jung et al. (54)	One group before and after	Community	Healthy men, 101 (100%), age (years): 58.9 ± 11.4	Information provision		0.86/low
Lee et al. (55)	Cross-sectional	Pilot project for peritoneal dialysis 8 hospitals	Patients with kidney failure, 101 (54.5%), age (years): 56.0	Information provision	SDM measurement scale	0.73/high
				Patient participation		
				Decision-making communication		

EORTC QLQ-INFO26, European Organization for Research and Treatment of Cancer quality of life questionnaire; RCT, randomized controlled trials; ROB, risk of bias; SDM, shared decision-making.

All studies assessed SDM entirely through patient self-reports, and most—except for two—did not utilize previously developed or validated theoretical models when designing the SDM components. These methodological features are not presented in Table 2 but were identified through a full-text review.

### 3.3 Outcomes for SDM effects

Outcomes were reported in 13 studies for affective–cognitive domains, in three studies for behavioral outcomes, and in three studies for health-related outcomes. Affective–cognitive effects included increased satisfaction, reduced decisional conflict, and enhanced knowledge. Patient satisfaction was examined in six studies, with five studies (83.3%) reporting significant improvement or high satisfaction, and one reporting no significant difference. Of the four studies that assessed decisional conflict, two studies (50%) reported a significant decrease, one study reported no significant difference compared to the non-SDM group, and one study did not provide sufficient evidence of a decrease (50%). The knowledge and attitude dimensions were assessed in five studies. Among them, three studies (60%) reported significant improvements or higher levels of help in terms of knowledge or experiential value, while two studies (40%) reported significant differences in knowledge and health literacy in the SDM group compared to other groups.

Behavioral outcomes showed mixed findings: two of the three studies (66.7%) reported improved willingness to act (30, 49), while one study (33.3%) reported a reduced intention to screen despite increased knowledge (54).

Health outcomes, including QoL, pain, and physical function, were reported in three studies. Two (66.7%) showed improvements in QoL or physical function (47, 49). One study reported that subjective pain improved with active patient participation, though expert-assessed physical signs showed no significant difference (53).

SDM-related outcomes were primarily assessed using self-reported measures. Measurement tools varied across studies, with some using the Consumer Assessment of Healthcare Providers and Systems, modified DCS items, or customized tools (Table 3).

**Table 3:** Outcomes for SDM effects.

Study, year	Cognitive outcomes			Behavioral outcomes	Clinical outcomes	Patients outcome evaluation tools
	Affective	Conflict	Knowledge/attitude			
Suh and Lee (28)	Patient satisfaction: +*					CAHPS
Min and Suh (46)	Patient satisfaction: +*					CAHPS
Nam et al. (47)^a^					Physical function: +*	DASH
Hwang et al. (29)	Patient satisfaction: *93% were satisfied*					One-item questions on satisfaction and respect respectively
	Feeling of respect: *97% felt respected*					
Yoon et al. (48)		Decisional conflict: ―*				DCS
Sim et al. (50)		Decisional conflict: *insufficient to decreased*				DCS
An et al. (49)^b^	Patient’s self-esteem: +*			Problem-solving ability: +*	Quality of life: +*	Self-Esteem Scale (69); Problem-Solving Inventory (70); WHOQOL-BREF
Gong et al. (26)^b^	Patient satisfaction: = *ns*	Decisional conflict: = *ns*	Knowledge: ≠*			DCS
Koo and Lee (51)		Decisional conflict: ―*				DCS
Kim (52)	Patient well-being: +*		Patient experiential value: +*			Sirgy’s items; EVS
Sohn et al. (30)^b^	Patient satisfaction: +*			Willingness to pay: +*		PROM(HIRA); one-item question on willingness to pay
Kang (53)^a^			Health literacy: ≠* in perceived roles		Subjective pain: ≠* in perceived roles	NVS for literacy; VAS and GCPS for subjective symptom; TMD specialist’s objective symptom assessment
					Objective signs: = *ns in perceived roles*	
Jung et al. (54)			Knowledge: +* Attitudes: Screening necessity: *+*	Intention to screen: ―*		10-item questions on knowledge; one-item questions on attitudes and intentions respectively
Lee et al. (55)	Patient satisfaction: o*ver 90% was satisfied*		Effects of SDM *over 90% helpful*			6-item patient satisfaction scale; One-item questions on effect

CAHPS, Consumer Assessment of Healthcare Providers and Systems; DASH, disabilities of the arm, shoulder, and hand; DCS, Decisional Conflict Scale; EVS, Experiential Value Scale; GCPS, Global Chronic Pain Scale; HIRA, Health Insurance Review and Assessment service; NVS, newest vital sign; PROM, patient-reported outcome measure; TMD: temporomandibular disorder; VAS, Visual Analog Scale; WHOQOL-BREF, World Health Organization Quality of Life Scale.

+, improved or increased; ―, decreased; *, significant; ≠, not equal; = ns, not significantly different.

a These studies reported results compared before and after.

b These studies reported outcomes based on comparisons with control groups.

### 3.4 Factors affecting patient outcome

Table 4 presents the relationships among the SDM components, participant characteristics, healthcare settings, and patient outcomes in each study.

**Table 4:** Impact of SDM programs, participants, and healthcare factors on patient outcomes.

Study, year	Affecting factors			Outcome and finding
	SDM components	Participants	Healthcare	
Suh and Lee (28)	Explanation of patients’ symptoms	Age, number of visits	Service type (outpatient and inpatient)	Opportunities to explain symptoms (*β* = 3.14, *p* < 0.01) and active patient involvement (*β* = 3.00, *p* < 0.01) in treatment decisions significantly increased satisfaction.
				Younger patients (*β* = −2.28, *p* < 0.05), and the number of visits (*β* = −2.76, *p* < 0.05) were negatively associated with satisfaction.
				Outpatients had significantly negatively associated satisfaction (*β* = −2.02, *p* < 0.05).
Min and Suh (46)	Sufficient explanation and Patient participation	Duration of military service	Service type (outpatient and inpatient)	Patients’ opportunity for symptom explanation and participation in treatment decisions were positively associated with satisfaction (physician, facilities and procedures, nurse, each *p* < 0.05).
				Longer military service and prior private hospital experience were associated with lower satisfaction (p < 0.05).
				Outpatients had significantly lower satisfaction compared to inpatients.
Nam et al. (47)	Consultation and decision-aid (IPDAS)	Patient’s preferred, experienced role	–	All groups significantly improved physical function (*p* < 0.001).
				Physical function: not significantly differ between groups (active, collaborate, passive, *p* = 0.770).
				Physical dysfunction (disability): matching of preferred and experienced role (14 ± 12) < more active role (22 ± 15, *p* = 0.049) or more passive role (22 ± 15, *p* = 0.032).
Hwang et al. (29)	Sufficient explanation, patient participation	Patient preference	–	Patient satisfaction: over 90% satisfied; feeling of respect: over 97% respected; 69.6% preferred a cooperative role, 18.3% preferred an active role and 12.1% preferred a passive role.
				Patients in active roles were significantly higher satisfaction (78.7%) and a greater sense of respect (76.6%) than those in collaborative or passive roles.
Yoon et al. (48)	Co-decision-making	Education level, family engagement	Physician’s sole decision	Family decision (*β* = 0.43, *p* < 0.001) or physician decision (*β* = 0.18, *p* = 0.008) significantly associated with decision conflict; shared decision-making between patient and doctor was negatively associated with decision conflict (*β* = −0.40, *p* < 0.001).
				Education level (*β* = −0.27, *p* < 0.001) were negatively associated with decision conflict.
Sim et al. (50)	Inadequate information	Income, education, sex, married, prognosis, treatment stage		Inadequate information provision: negative association with high decision conflict (OR, 0.43; 95% CI 0.26–0.71) and decision uncertainty (OR, 0.46; 95% CI 0.27–0.77).
				Inadequate information factors: lower income and education, women, unmarried, good prognosis, early stage of treatment (*p* < 0.05).
An et al. (49)	SDM training: education, decision aids, role-playing (psychosocial rehabilitation model)^a^	–	SDM team providers	Self-esteem, problem-solving ability, quality of life: case group > Control group (*p* < 0.05), with significantly greater pre-post changes (*p* < 0.05).
				Psychiatric practitioner (SDM administrator), mental health nurse (SDM assistants), attending physician (decision-making and feedback).
Gong et al. (26)	Decision-aid: 6 min videoclip (IPDAS)	Knowledge, symptom	–	CTS knowledge: case > control (7.4 vs. 6.6; *p* = 0.04).
				Patient satisfaction and decisional conflict: case vs. control (no differences).
				Less severe symptoms had greater decisional conflict (*r* = −0.29, *p* = 0.02).
Koo and Lee (51)	Explanation of patients’ health problems and decision-making	Preference for quality of life, anxiety	–	SDM (*β* = −0.25, *p* < 0.001) had a greater impact on decisional conflict in pre-surgery cancer compared to other factors.
				Higher patient preference for quality of life significantly decreased decision conflict (*r* = −0.28, *p* = 0.003).
				As patient anxiety increased, the level of decision conflict increased (*r* = 0.28, *p* = 0.003).
Kim (52)	Patient participation	Satisfaction with life	Physician resource	Patient participation significantly increases in patients’ experiential value (*β* = 0.661, *p* < 0.01) and perceived well-being (*β* = 0.261, *p* < 0.05).
				Patient satisfaction with life was positively associated with patients’ perceived well-being (*β* = 0.633, *p* < 0.01).
				Physician’s affective and cognitive resource had a positive effect on patient participation (*β* = 0.263, *β* = 0.508, *p* < 0.05).
Sohn et al. (30)	In-depth consultations: over 15 min	–	Medical departments	In-depth consultations resulted in positive outcomes for patient-centeredness and patient satisfaction (*p* < 0.001).
				The increase in patient satisfaction was highest in internal medicine, followed by surgery and pediatrics.
				Physician questionnaire: physicians reported improved satisfaction after in-depth consultations, with 92% showing intent to participate in the future.
Kang (53)	Patient participation	Education level, health literacy, perceived patient role	–	Education level is significantly associated with perceived role (*p* = 0.023).
				Adequate health literacy is significantly associated with higher perceived participation (*p* = 0.002).
				Subjective pain (worsening): active and collaborate patient’ role < passive patient’ role, (*p* < 0.001).
				Objective signs by specialist did not significant differences between perceived role.
Jung et al. (54)	Web-based decision-aid (IPDAS)	Prior screening experience	–	Knowledge significantly increased: before vs. after (6.85 ± 1.03 vs. 7.57 ± 1.25, *p* < 0.001); negative attitude toward screening: no difference (*p* = 0.564); intention to not screen: significantly increased from 27.7 to 51.5% (*p* < 0.001).
				Subgroup analysis (screened/non-screened): knowledge significantly increased in the non-screened group (*p* < 0.001), but not in the screened group; Intentions not to screen increased significantly in both groups (*p* < 0.007).
Lee et al. (55)	Decision-aid and consultation, decision coaching (three-talk model)	Dialysis methods	–	Over 90% were satisfied with the SDM process for selecting dialysis methods, and 92.1% reported it was helpful.
				Peritoneal dialysis showed higher positive outcomes than hemodialysis in co-selection making (94% vs. 87.5%, *p* = 0.012) and agreement on the treatment process (97% vs. 81.3%, *p* = 0.058). Satisfaction with the SDM process was also higher for peritoneal dialysis (*p* < 0.05).

a The Psychosocial Rehabilitation Model was developed by Hamann et al. (71) and subsequently revised by Korean Association for Psychosocial Rehabilitation (KAPR) (72).

#### 3.4.1 SDM component factors

The studies included in this review implemented a variety of SDM programs, ranging from decision-aid tools to multi-week patient education sessions. A common SDM component in South Korean healthcare, identified across multiple studies, involved consultations with healthcare providers, particularly those emphasizing explanations of health problems or symptoms (28–30, 46, 51).

Five of the 14 studies investigated the effects of using theoretical models, and certified decision aids to conduct SDM in clinical practice and guide complex decision-making (26, 47, 49, 54, 55). Two studies using only decision aids reported no significant differences in outcomes, including satisfaction, decisional conflict (26), or negative attitudes and intentions (54), except for increased knowledge. However, one study consistently showed that pairing decision aids with sufficient explanation or counseling from healthcare professionals led to improved patient-centeredness and other subjective health outcomes (47).

Furthermore, interventions that integrated counseling, education, and decision aids based on theoretical SDM models—encompassing information provision, communication, patient participation, and decision-making—demonstrated improvements across multiple patient outcomes, including problem-solving ability, willingness to pay, and intent to undergo screening (49, 55).

#### 3.4.2 Participant factors

Twelve studies evaluating the impact of SDM implementation on patient outcomes considered patient factors as mediating or moderating variables. These factors included demographic characteristics, health status, attitudes, preferences, and experience with SDM programs.

Several studies have found that participants’ positive attitudes, preferences, and perceived patient roles were associated with positive outcomes (29, 47, 51–53). Conversely, negative outcomes related to attitudes and intentions toward screening were linked to prior experiences with screening (54).

Two studies found that factors such as severe disease symptoms and lower educational levels negatively impacted decisional conflict (26, 48), while vulnerable groups—including those with lower income or education, women, and unmarried individuals—perceived the provided information as inadequate, leading to increased decisional conflict (50). In contrast, Kang (53) found that higher education levels and stronger health literacy significantly enhanced patients’ perceived roles and levels of participation, thereby improving their subjective health outcomes.

#### 3.4.3 Healthcare factors

Six studies evaluated the association between SDM programs and healthcare factors and outcomes (28, 30, 46, 48, 49, 52). Two studies reported that outpatient settings yielded higher satisfaction outcomes than inpatient settings (28, 46). Sohn et al. (30) showed increased patient-centeredness and patient satisfaction with in-depth consultations lasting over 15 min, with satisfaction highest in internal medicine departments. Yoon et al. (48) reported that family engagements were higher than sole engagements, which were significantly associated with decision conflicts. An et al. reported significant improvements in patient outcomes when several mental healthcare professionals participated in SDM and collaborated as a team (49). Kim (52) found that the provision of emotional and cognitive resources by physicians in SDM encourages patient participation, improving their perceived well-being and experiential value. These results suggest that SDM is an effective mechanism for influencing outcomes by enhancing patient attitudes and facilitating behavioral change.

### 3.5 Quality assessment

**Figure 2:**
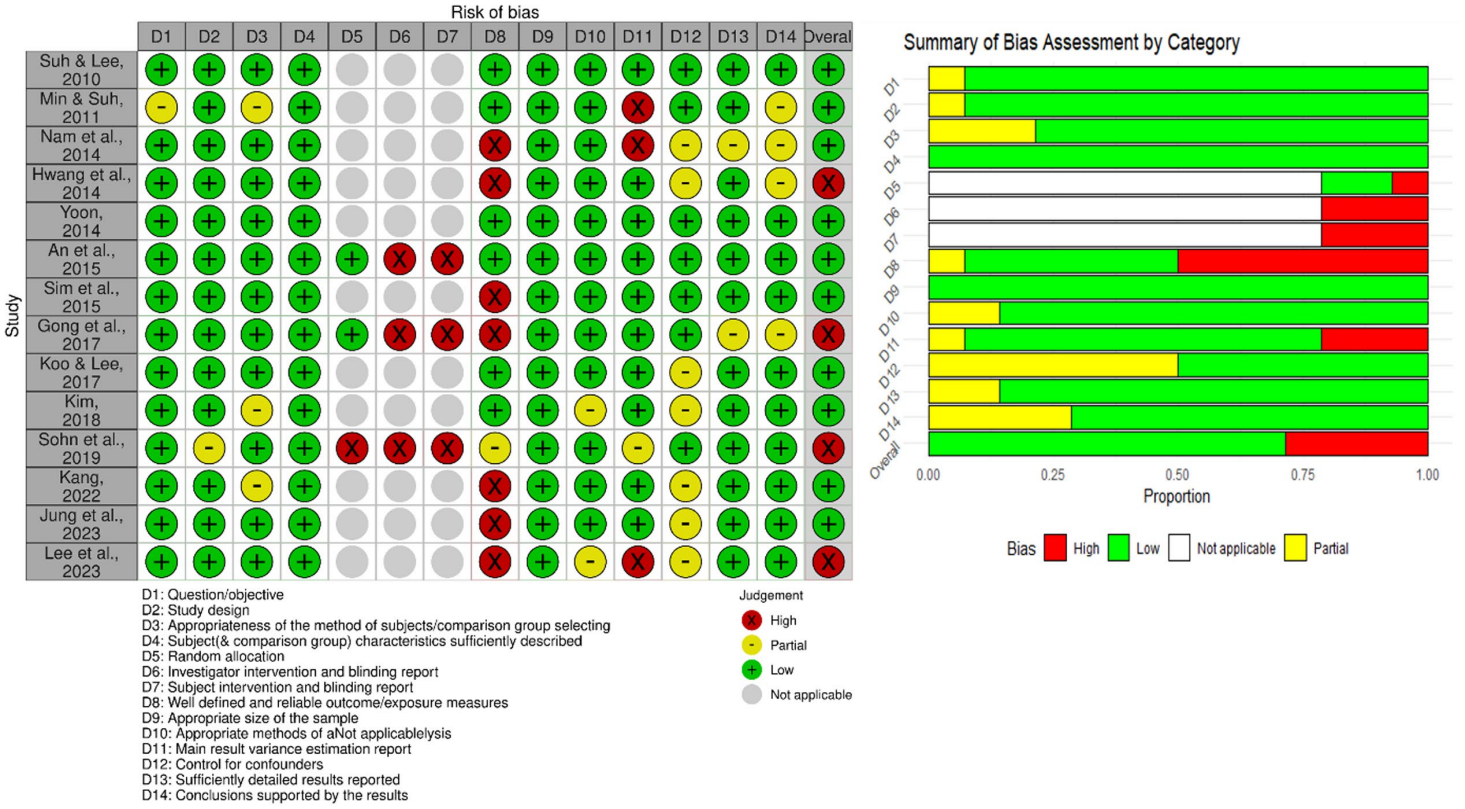
Quality assessment summary.

Quality assessment using the QualSyst tool indicated that 10 studies had a low risk of bias, while 4 showed a higher risk. Only two studies fully met the quality criteria across all domains. The QualSyst tool does not provide explicit exclusion criteria or guidance on handling studies at high risk of bias, noting that inclusion decisions remain at the researcher’s discretion after calculating the score. In our study, the lowest risk-of-bias score was 0.68, with a relatively generous threshold of 0.55 applied to include studies in the systematic review (45). Therefore, all studies were retained in the analysis. A summary of the quality assessment is provided in Figure 2 (56).

## 4 Discussion

This systematic review synthesized 14 empirical studies on SDM in South Korea, evaluating their implementation across diverse clinical settings and their effects on patient outcomes. While most studies addressed individual elements such as information provision and patient participation, only a few incorporated all core SDM components—namely, information provision, patient participation, communication, and decision-making—or were guided by established theoretical frameworks. These findings suggest that SDM may improve patient outcomes, although limitations in intervention delivery and outcome assessment remain widespread.

Consistent with trends in the international literature (57), SDM interventions in South Korea have been most frequently applied in oncology and chronic care settings, where treatment decisions often involve multiple viable options and require value-sensitive trade-offs that are best addressed by incorporating patients’ unique values and preferences (58).

Cognitive outcomes were the most commonly assessed across the included studies, encompassing satisfaction, decisional conflict, knowledge, and attitudes. Consistent with previous reviews (58, 59), SDM was positively associated with improvements in affective–cognitive outcomes such as increased patient satisfaction, enhanced knowledge, and reduced decisional conflict. However, some studies reported no association or even negative effects, depending on the SDM components, types of decision aids used, and participant characteristics—indicating inconsistencies in the overall evidence.

Several factors may explain the inconsistencies in patient outcomes observed across studies. A key contributor is the heterogeneity of SDM interventions. Studies varied substantially in the SDM components they included, ranging from primarily providing information to integrating more comprehensive elements such as patient participation, shared deliberation, and decision aids. Consistent with reviews from other countries facing similar challenges in early SDM implementation (18, 19), this study highlights a limited understanding of the SDM concept in South Korea. Second, differences in outcome measurement across studies could have contributed to the inconsistent results. Studies used a variety of instruments to measure cognitive outcomes, including satisfaction and decisional conflict. This underscores the need for reliable and standardized instruments to enable consistent assessment and foster a shared framework among researchers and healthcare professionals (60). Third, methodological ambiguities or design limitations, including small sample sizes, a lack of control groups, or high attrition rates, may have affected the validity and reliability of the findings (61). These limitations make it difficult to draw firm conclusions about the effectiveness of SDM interventions and highlight the need for future research employing more rigorous designs, such as adequately powered randomized controlled trials with appropriate controls.

This study found that PDAs alone are insufficient to reduce decisional conflict or improve negative attitudes toward cancer screening. Sole reliance on PDAs neither reduced decisional conflict (26) nor influenced negative attitudes toward cancer screening (54). While prior systematic reviews have highlighted the effectiveness of decision support tools in improving patient outcomes, they have also noted that their use does not guarantee the occurrence of SDM, as evidenced in this review (32). Similarly, another literature review suggested that PDAs can be difficult for patients to understand and access due to their limited availability, outdated information, and poor discoverability (62). In contrast, studies employing structured training formats—such as a psychosocial rehabilitation program with role-playing and simulation (49) or decision support and coaching based on the Three-Talk Model (55)—have demonstrated improvements across multiple patient outcomes, suggesting that SDM programs grounded in theoretical frameworks and incorporating essential interactive components can be effective. This is supported by findings that patient outcomes improved when PDAs were combined with education and consultation from healthcare professionals (47, 48). However, such comprehensive approaches remain limited in South Korea. Moreover, only three studies explicitly mentioned using the IPDAS criteria to evaluate their tools (26, 47, 54). Given the absence of IPDAS guidelines reflecting Korean culture and language, there is an urgent need to develop these guidelines and design a patient decision aid development process based on them. Therefore, future SDM interventions in South Korea should focus on integrating PDAs into more comprehensive, interactive programs that incorporate elements of education, counseling, and shared deliberation. Efforts should also be directed toward developing culturally and linguistically appropriate IPDAS guidelines.

Most studies have examined the variables that moderate the effects of SDM on patient outcomes. In the patient domain, factors such as income, educational level, illness stage, health literacy, and patient attitudes influenced outcomes (26, 28, 48, 50, 52–54). Previous reviews reported similar findings regarding the influence of age and educational level (63). Older patients have limited clinical knowledge and lower overall educational levels, which may explain their tendency to take a more passive role in decision-making compared to younger patients. A study conducted in Western countries found that older adults expressed a desire to share or make their own medical decisions (64).

Enhancing patients’ preferences (29, 47), self-efficacy (49), and health literacy (53) can strengthen their motivation to participate and increase satisfaction with care. These findings suggest that SDM is an effective mechanism for improving patient attitudes and fostering behavioral changes, ultimately leading to better patient outcomes.

Education and training for patients and healthcare professionals are essential for implementing SDM in healthcare settings. Although this study identified only a limited positive impact of education and training on SDM outcomes, previous reviews have shown that SDM training can improve clinicians’ attitudes and knowledge, enhance patient–doctor communication, and positively influence patients’ QoL and SDM skills (57, 65, 66).

The present review identified factors, including inpatient services and physician support, as critical considerations (50, 52, 53). Lower SDM effectiveness among outpatients may stem from the structural challenges in South Korea’s healthcare system. For effective SDM, sufficient consultation time is crucial; however, limitations in South Korea’s healthcare delivery system and low reimbursement rates result in severely restricted outpatient consultation times (30). Therefore, active physician support is vital for fostering a high-quality patient–physician relationship and significantly improving patient participation (63, 64).

Patient characteristics, prior experience, health literacy, preferences, and perceptions are significant factors in determining the successful implementation of SDM. SDM providers should carefully consider these factors when exchanging information and engaging in patient decision-making.

Although SDM conceptually integrates multiple components—including information provision, patient participation, communication, and deliberation—the relevance of each element may vary by clinical context, disease type, and healthcare delivery environment. In the South Korean healthcare system, with time-constrained outpatient consultations and limited reimbursement mechanisms, applying the full SDM model uniformly across all diseases and patient groups may be impractical.

Evidence from reviewed studies highlights that the effectiveness of SDM varies depending on the clinical context and the complexity of the decisions involved. In emotionally charged or preference-sensitive conditions such as oncology and dialysis, interventions that integrate communication, counseling, and deliberation are particularly critical to supporting patient engagement and value-based decision-making. By contrast, in lower-stakes decisions, providing structured information with minimal interaction may be sufficient.

Moreover, patient characteristics—such as age, health literacy, and prior experience with SDM—necessitate tailored communication approaches. For example, older adults and individuals with limited health literacy often benefit more from guided participation than from passive information delivery (64). Conversely, younger or more health-literate patients may respond better to tools that emphasize autonomy and provide accessible information (67).

These differentiated approaches suggest the need for a contextualized SDM framework in South Korea—one that identifies essential or optional SDM components based on the clinical setting, patient capacity, and decision complexity. Developing differentiated models based on clinical need and structural feasibility could enhance SDM implementation in the South Korean context while preserving its patient-centered benefits. This would also support national policy efforts to integrate SDM into routine care delivery without overburdening the system.

### 4.1 Strengths and limitations

The strength of our review lies in being the first to comprehensively identify and synthesize the literature on SDM programs implemented in the South Korean healthcare environment and their associated measured outcomes. This review provides a detailed analysis of the content of SDM interventions and the factors influencing patient outcomes from both the patient and healthcare provider perspectives. By examining these key elements, this review provides valuable insights into the effectiveness of SDM in the South Korean context. These insights can serve as a basis for developing future healthcare policies, guidelines, and programs to enhance patient-centered care and decision-making in South Korea. Moreover, the findings may also inform similar efforts in other countries with comparable healthcare system constraints.

This study had some limitations. Most studies did not report the interventions’ qualitative fidelity, limiting our ability to assess the quality of SDM processes and patient–physician interaction dynamics. Consequently, how effectively the SDM components were implemented and how they influenced the observed outcomes remains unclear. While a meta-analysis could have increased the statistical power for effect sizes, it was not feasible due to the heterogeneity of the interventions and outcomes, as well as the limited number of studies available. Instead, the findings were synthesized using a narrative approach. The diverse outcome measures across studies make comparing decision aids at the outcome level challenging, thereby adding complexity to the analysis. Given these limitations and the field’s current state, our findings should be viewed as preliminary. Nevertheless, they highlight practical challenges and emerging strategies that are relevant to global discussions on SDM institutionalization and implementation across diverse healthcare systems.

## 5 Conclusion

This review provides a comprehensive overview of shared decision-making (SDM) programs and their effectiveness in the South Korean healthcare environment, demonstrating the potential for SDM to be implemented across diverse clinical settings. While most studies suggest a positive association between SDM and improvements in patient knowledge, reduced decisional conflict, and increased satisfaction, these outcomes have not been consistently observed across all cases. Therefore, maximizing the benefits of SDM and tailoring implementation strategies to the unique South Korean healthcare landscape will require further research and concerted policy initiatives.

Specifically, future efforts must prioritize developing a South Korea-specific SDM model that reflects the characteristics of the Korean healthcare system—notably its brief consultation times, limited reimbursement for counseling, and persistent provider–patient information asymmetry—as well as its prevailing cultural values, rather than relying on uniform SDM models. This model should incorporate clinical area specialization, customizing SDM to address specific contexts such as chronic disease management, cancer treatment, and mental health services, while remaining sensitive to the needs of particular patient groups, including older adults and those with limited health literacy. For older adults, respecting family-centered decision-making processes is essential, and providing easy-to-understand language and visual aids is crucial for those with lower health literacy. Simultaneously, research should explore effective strategies for integrating these elements into existing healthcare workflows, potentially through pre-visit information preparation and the use of online decision support tools to facilitate more efficient consultations.

Alongside model development, the consistent and reliable measurement of SDM effectiveness will depend on the development and application of standardized evaluation tools. These tools should encompass measurement domains that capture a range of outcomes, incorporating patient knowledge, decisional conflict, satisfaction levels, quality of life, behavioral changes like treatment adherence, and objective health indicators. Cultural appropriateness must be ensured by either developing evaluation tools specifically for the Korean cultural context or rigorously adapting existing tools through careful translation and validation. These measurement efforts should be accompanied by rigorous study designs and concurrent qualitative research to definitively establish the effectiveness of SDM interventions. Future research should employ RCTs and quasi-experimental designs, complemented by qualitative approaches that allow for a nuanced understanding of patient and healthcare professional experiences, as well as the qualitative aspects of the SDM process.

Furthermore, policy support and incentives are essential for promoting and encouraging SDM implementation. Financial incentives could be considered to integrate SDM into value-based payment models or bundled payment agreements, as well as to provide direct reimbursement for the time invested in SDM activities.

In conclusion, successful SDM integration in Korea hinges on the systematic and pragmatic development of a comprehensive framework that incorporates key components, such as validated conversation models, a certification/accreditation system for decision support tools, and education and training programs, alongside a careful exploration of its suitability for specific clinical settings or patient populations in the Korean context.
