# Supplementary material for: A systematic review of the effects of shared decision-making in the South Korean healthcare system
Source: Front Public Health. 2026 Jan 5;13:1667803. doi: 10.3389/fpubh.2025.1667803 (PMC12812929; doi:10.3389/fpubh.2025.1667803)
Supplement: Supplementary file 1 [file Supplementary_file_1.docx]

Supplementary Material

# Appendix A: Overview of inclusion and exclusion criteria

***Include***

1. Studies published up to July 2024 that examine the effectiveness of implementing and disseminating shared decision-making (SDM) systems include studies in which the terms “shared decision-making” or “participatory decision-making” were explicitly used in the intervention description, or where it was indicated that the intervention was designed to implement a shared decision-making process between clinicians and patients.

2. Studies involving adults aged 18 years or older who are capable of making decisions about various healthcare services.

3. Studies focusing on South Korean individuals who received services in healthcare institutions in South Korea.

4. Studies focusing on the potential impact and effectiveness of SDM interventions on cognitive, behavioral, or clinical outcomes, as well as patient experiences.

5. Randomized controlled trials, non-randomized controlled trials (e.g., pre-post studies, studies with or without contemporary controls), and observational studies (e.g., cohort studies, case-control studies, cross-sectional studies).

***Exclude***

1. Surrogate decision-making owing to being aged younger than 18 years or lacking decision-making capacity.

2. Studies involving foreigners living in South Korea or South Koreans living abroad.

3. Studies on life-sustaining or end-of-life decisions, such as advance directives.

4. Studies focused on the validation or reliability of SDM/PDA tools or SDM/PDA assessment instruments (e.g., reporting and measurement tools).

5. Studies that did not report predefined outcomes or did not quantitatively assess these outcomes.

6. Studies published in languages other than Korean or English.

# Appendix B: Search strategy

Supplementary Table 1. PubMed

| ECLIPSE | # | Query | Results |
| --- | --- | --- | --- |
| [E] | 1 | "shared decision making"[Title/Abstract] OR "decision making, shared"[MeSH Terms] | 17,026 |
|  | 2 | ("shared"[Title/Abstract] OR "joint"[Title/Abstract] OR "participatory"[Title/Abstract]) AND "decision making"[Title/Abstract] | 25,548 |
|  | 3 | ("medical"[Title/Abstract] OR "treatment"[Title/Abstract] OR "clinical"[Title/Abstract]) AND "decision"[Title/Abstract] | 220,741 |
|  | 4 | "patient participation"[MeSH Terms] OR "patient participation"[Title/Abstract] | 32,738 |
|  | 5 | "Patient-Center*"[Title/Abstract] OR (("patient*"[Title/Abstract] OR "patients"[MeSH Terms]) AND "center*"[Title/Abstract]) | 528,628 |
|  | 6 | 1 OR 2 OR 3 OR 4 OR 5 | 764,717 |
| [L] | 7 | "Korea*"[MeSH Terms] OR "Korea*"[All Fields]) | 630,811 |
| [E] and [C] and [L] | 8 | 1 AND 7 | 18,105 |
|  | 9 | 8 Filters: Humans, English, Korean, Adult: 19+ years | 8,921 |

# Supplementary Table 2. Embase

| **Embase** | | | |
| --- | --- | --- | --- |
| ECLIPSE | # | Query | Results |
| [E] | 1 | shared decision making.mp. or *decision making/ or *patient participation/ or *shared decision making/ | 110,976 |
|  | 2 | participatory decision making.mp. | 254 |
|  | 3 | joint decision making.mp. | 528 |
|  | 4 | *clinical decision making/ or *medical decision making/ or medical decision.mp. | 110,436 |
|  | 5 | Patient-Centeredness.mp. | 1,938 |
|  | 6 | 1 OR 2 OR 3 OR 4 OR 5 | 219,936 |
| [L] | 7 | Korea*.mp. or exp Korea/ or exp South Korea/ | 189,508 |
| [E] and [C] and [L] | 8 | 6 AND 7 | 705 |
|  | 9 | limit 8 to (human and (English or Korean) and (adult <18 to 64 years> or aged <65+ years>)) | 373 |

# Supplementary Table 3. Cochrane

| **Cochrane** | | | |
| --- | --- | --- | --- |
| ECLIPSE | # | Query | Results |
| [E] | 1 | ("shared decision-making"):ti,ab,kw | 2,347 |
|  | 2 | MeSH descriptor: [Decision Making, Shared] 2 tree(s) exploded | 203 |
|  | 3 | (joint decision making):ti,ab,kw | 647 |
|  | 4 | (medical decision making):ti,ab,kw | 7,940 |
|  | 5 | (clinical decision making):ti,ab,kw | 16,899 |
|  | 6 | (participatory decision making):ti,ab,kw | 143 |
|  | 7 | MeSH descriptor: [Patient Participation] 3 tree(s) exploded | 2,216 |
|  | 8 | (patient participation):ti,ab,kw | 233,087 |
|  | 9 | (Patient-Centeredness):ti,ab,kw | 174 |
|  | 10 | #1 OR #2 OR #3 OR #4 OR #5 OR #6 OR #7 OR #8 OR #9 OR #10 | 245,916 |
| [L] | 11 | (Korea*):All Text | 27,296 |
|  | 12 | MeSH descriptor: [Republic of Korea] explode all trees | 1,873 |
|  | 13 | #11 OR #12 | 27,326 |
| [E] and [L] | 14 | #10 AND #13 | 3,681 |
|  | 15 | Trial matching #14 , Language: Korean, English | 3,204 |

# Supplementary Table 4. Web of science

| **Web of science** | | | |
| --- | --- | --- | --- |
| ECLIPSE | # | Query | Results |
| [E] | 1 | TS=(shared decision making) | 59,309 |
|  | 2 | (((TS=(participatory decision making)) OR TS=(joint decision making)) OR TS=(medical decision making)) OR TS=(clinical decision making) | 176,302 |
|  | 3 | #1 OR #2 | 217,781 |
| [L] | 4 | ALL=(Korea*) | 1,978,713 |
| [E] and [L] | 5 | #3 AND #4 | 3,867 |
|  | 6 | #5 AND (LA=(English) OR LA=(Korean)) | 2,872 |

# Supplementary Table 5. ScienceON

| **#** | **검색어** | **검색결과** | |
| --- | --- | --- | --- |
| 1 | 논문명=공유 의사결정 OR 논문명=공동 의사결정 OR 논문명=함께하는 의사결정 | 64 | |
| 2 | 초록=공유 의사결정 OR 초록=공동 의사결정 OR 초록=함께하는 의사결정 | 550 | |
| 3 | 주제어=공유 의사결정 OR 주제어=공동 의사결정 OR 주제어=함께하는 의사결정 | 19 | |
| 4 | 논문명=진료 의사결정 OR 초록=진료 의사결정 OR 주제어=진료 의사결정 OR 논문명=치료 의사결정 OR 초록=치료 의사결정 OR 주제어=치료 의사결정 | 197 | |
| 5 | 논문명=환자참여 OR 초록=환자참여 OR 주제어=환자참여 OR 논문명=환자중심의료 OR 초록=환자중심의료 OR 주제어=환자중심의료 | 1242 | |
| **합계** | | **2072** |  |

# Supplementary Table 6. RISS

| **#** | **검색어** | **검색결과** |
| --- | --- | --- |
| 1 | 논문명 : 공유 의사결정 <OR> 논문명 : 공동 의사결정 <OR> 논문명 : 함께하는 의사결정 | 95 |
| 2 | 초록 : 공유 의사결정 <OR> 초록 : 공동 의사결정 <OR> 초록 : 함께하는 의사결정 | 1413 |
| 3 | 주제어 : 공유 의사결정 <OR> 주제어 : 공동 의사결정 <OR> 주제어 : 함께하는 의사결정 | 90 |
| 4 | 논문명 : 진료 의사결정 <OR> 초록 : 진료 의사결정 <OR> 주제어 : 진료 의사결정 <OR> 논문명 : 치료 의사결정 <OR> 초록 : 치료 의사결정 <OR> 주제어 : 치료 의사결정 | 792 |
| 5 | 논문명 : 환자참여 <OR> 초록 : 환자참여 <OR> 주제어 : 환자참여 <OR> 논문명 : 환자중심의료 <OR> 초록 : 환자중심의료 <OR> 주제어 : 환자중심의료 | 2775 |
| **합계** | | **5165** |

# Supplementary Table 7. Kmbase

| **#** | **검색어** | **검색결과** |
| --- | --- | --- |
| **1** | (([TITLE=공유 의사결정] OR [TITLE=공동 의사결정]) OR [TITLE=함께하는 의사결정]) | 6 |
| **2** | (([ABSTRACT=공유 의사결정] OR [ABSTRACT=공동 의사결정]) OR [ABSTRACT=함께하는 의사결정]) | 5 |
| **3** | (([KEYWORD=공유 의사결정] OR [KEYWORD=공동 의사결정]) OR [KEYWORD=함께하는 의사결정]) | 6 |
| **4** | ((((([TITLE=진료 의사결정] OR [ABSTRACT=진료 의사결정]) OR [KEYWORD=진료 의사결정]) OR [TITLE=치료 의사결정]) OR [ABSTRACT=치료 의사결정]) OR [KEYWORD=치료 의사결정]) | 10 |
| **5** | ((((([TITLE=환자참여] OR [ABSTRACT=환자참여]) OR [KEYWORD=환자참여]) OR [TITLE=환자중심의료]) OR [ABSTRACT=환자중심의료]) OR [KEYWORD=환자중심의료]) | 23 |
| **합계** | | **50** |

# Appendix C: Exclusion criteria

Supplementary Table 8**.** Excluded studies and reasons for exclusion

| **No.** | **Author(s)** | **Year** | **Title** | **Journal** | **Volume**  **(issue)** | **Reason for exclusion** |
| --- | --- | --- | --- | --- | --- | --- |
| 1 | Park, Chang-Gi; Kim, Gong-Hyeon; Hwang, In-Gyeong; Kim, Yong-Wan | 1992 | 환자가 인지하는 병원의 의료기술외적 서비스가 환자의 병원 재이용 의사결정에 미치는 영향에 관한 조사연구 | Journal of the Korean Hospital Association | 21(7) | Not pre-discussed intervention |
| 2 | Song, Si Eun; Li, Seung Won; Cho, Kyoo Sung; Chai, Jung Kiu; Kim, Chong Kwan | 1998 | Decision Making on the Non-surgical, Surgical Treatment on Chronic Adult Periodontitis | Journal of Periodontal & Implant Science | 28(4) | Not pre-discussed intervention |
| 3 | Yi, Myungsun | 2003 | Decision-making process for living kidney donors | Journal of nursing scholarship | 35(1) | Not pre-discussed designs |
| 4 | Oh, Do-Youn; Kim, Jee-Eun; Lee, Chee-Hun; Lim, Jae-Sung; Jung, Kyung-Hye; Heo, Dae Seog; Bang, Yung-Jue Bang; Kim, Noe Kyeong | 2004 | Discrepancies among patients, family members, and physicians in Korea in terms of values regarding the withholding of treatment from patients with terminal malignancies | Cancer | 100(9) | Not pre-discussed intervention |
| 5 | An, Kyung-Eh; Kim, Jeong-Eun; Kang, Kim Min-Ah; Jung, Yoen-Yi | 2006 | Reducing Medical Errors: Patients' Self Protect Behaviors and Involvement in Decision Making | Korean Journal of Health Policy & Administration | 16(3) | Not pre-discussed intervention |
| 6 | Hwang, Sun-Wook; Choi, Chang-Jin | 2006 | Patient-centered Attitudes in Primary Care Patients | Korean Journal of Family Medicine | 27(12) | Not pre-discussed outcomes |
| 7 | Lee, Sang-Mok | 2007 | A Cross-Cultural Approach to Biomedical Ethics: Medical Decision Making | Korean Journal of Medical Ethics | 10(1) | Not pre-discussed intervention |
| 8 | Kim, Jin Kyong | 2008 | Shared Decision-Making as a Model of Medical Decision-Making | Korean Journal of Medical Ethics | 11(2) | Not pre-discussed intervention |
| 9 | Nam, Tae-Hee | 2008 | A Study on the medical decision making for an optimum medical treatment | Journal of the Korea Computer Industry Society | 9(1) | Not pre-discussed participants |
| 10 | Chang, Soo Jung; Lee, Kyung Ja; Kim, In Sook; Won Hee, Lee | 2008 | Older Korean people's desire to participate in health care decision making | Nursing Ethics | 15(1) | Not pre-discussed participants |
| 11 | Lee, Byoung Kwan; Byoun, Woong Jun; Lim, Ju Lee | 2010 | The Influence of Individual’s E-Health Literacy on Doctor-Patient Communication | Journal of Cybercommunication Academic Society | 27(3) | Not pre-discussed participants |
| 12 | Jo, Kae-Hwa | 2010 | Nurse's Conflict Experience toward End-of-life Medical Decision-making | Korean Journal of Adult Nursing | 22(5) | Not pre-discussed designs |
| 13 | Lee, Myung Kyung; Noh, Dong Young; Nam, Seok Jin; Ahn, Se Hyun; Park, Byeong Woo; Lee, Eun Sook Lee & Yun, Young Ho | 2010 | Association of shared decision-making with type of breast cancer surgery: a cross-sectional study | BMC Health Service Research | 10 | Not pre-discussed designs |
| 14 | 김성근 | 2010 | 말기환자 진료에 있어 의사결정에 대한 의료 윤리적 고찰: 의사, 환자, 환자가족 공동의사결정을 중심으로 | The Catholic Thought | - | Not pre-discussed designs |
| 15 | Jo, Kae Hwa; An, Gyeong Ju; Kim, Gyun Moo | 2011 | A Factor Analysis of the Impediments to End-Stage Medical Decision-Making as Perceived by Nurses and Physicians in South Korea | Korean Journal of Medical Ethics | 14(4) | Not pre-discussed designs |
| 16 | Kim, Hyoung Suk | 2011 | The Review for the Problem with Decision-Making in Medical Context: Focusing on Concept of Autonomy | Personalism Bioethics | 1(1) | Not pre-discussed intervention |
| 17 | Shin, Dong Wook; Kim, So Young; Cho, Juhee; Robert W. Sanson-Fisher; Eliseo Guallar; Chai, Gyu Young; Kim, Hak-Soon; Park, Bo Ram; Park, Eun-Cheol; Park, Jong-Hyock | 2011 | Discordance in Perceived Needs Between Patients and Physicians in Oncology Practice: A Nationwide Survey in Korea | Journal of Clinical Oncology | 29(33) | Not pre-discussed outcomes |
| 18 | Jo, Kae-Hwa; Kim, Yeon Ja; Shon, Ki Cheul | 2012 | Types of Perception toward End-of-Life Medical Decision-making of Clinical Nurses | Journal of Hospice and Palliative Care | 15(1) | Not pre-discussed participants |
| 19 | Jo, Kae Hwa | 2012 | Development and Evaluation of Shared Medical Decision-Making Scale for End-of-Life Patients in Korea | Journal of Korean Academy of Nursing | 42(4) | Not pre-discussed intervention |
| 20 | D. Heo; J. K. Lee; A. An; B. Keam; T. Kim; S. Lee; D. Kim | 2012 | Factors associated with surrogate decision-making in advanced cancer patients: A longitudinal study | Annals of Oncology | 9) | Not pre-discussed outcomes |
| 21 | Lee, Hyuk; Lee, Yong Chan; Shin, Suji Shin; Park, Jun Chul; Shin, Sung Kwan; Lee, Sang Kil; Noh, Sung Hoon | 2012 | Participation and conflict in the decision-making process for endoscopic resection or surgical gastrectomy for early gastric cancer | Journal of Surgical Oncology | 106(1) | Not pre-discussed designs |
| 22 | Lee, Hew-Jeong & Yang, Jin-Hyang | 2013 | Factors Influencing Older Patients' Participation in Decision Making Regarding Cancer Surgery | Journal of Korean Gerontological Nursing | 15(1) | Not pre-discussed intervention |
| 23 | Jo, Kae-Hwa & An, Gyeong-Ju | 2013 | Factors affecting shared decision making at end of life in Korean adults | Holistic Nursing Practice | 27(6) | Not pre-discussed outcomes |
| 24 | Lee, Seung Mi; Nam, Hyun Woo; Kim, Eun Na; Shin, Dong Wook; Moon, Hye-Jin; Jeong, Joo Yeon; Kim, Su Ah; Kim Byoung Jae; Lee, Sang Kun; Jun, Jong Kwan | 2013 | Pregnancy-related knowledge, risk perception, and reproductive decision making of women with epilepsy in Korea | Seizure | 22(10) | Not pre-discussed designs |
| 25 | Lee, Jong-Hak & Kim, Chan-Jung | 2013 | Effect of Doctors' Patient-centered Communication on the Patient Satisfaction and Treatment Outcomes: Focusing on Mediating Effect of Patient Participation | The Journal of the Korea Contents Association | 13(11) | Not pre-discussed intervention |
| 26 | Lee, Jong-Hak & Kim, Chan-Jung | 2013 | Effect of Patient-centered Communication of Doctor on Patient Participation: Focusing on Moderating Effect of Trust | The Journal of the Korea Contents Association | 13(3) | Not pre-discussed outcomes |
| 27 | Jo, Kae Hwa & Kim, Gyun Moo | 2014 | Types of Shared Medical Decision Making for Terminally Ill Patients | Journal of Hospice and Palliative Care | 17(4) | Not pre-discussed designs |
| 28 | Lee, Eun-Young | 2014 | A Study of Patient Decision Aids (PtDAs) for Shared Decision Making in Medical Decisions | Bioethics Policy Studies | 8(1) | Not pre-discussed designs |
| 29 | Park, Seon Gyu | 2014 | Shared decision support system on dental restoration | Journal of Korean Academy of Dental Administration | 2(1) | Not pre-discussed intervention |
| 30 | Kan, Soo Jin; Lee, Tae Wha; Michael K. Paasche-Orlow; Kim,Gwang Suk; Won, Hee Kwan | 2014 | Development and Evaluation of the Korean Health Literacy Instrument | Journal of Health Communication | 19 | Not pre-discussed designs |
| 31 | Ahn, Joon Tae; Park, Gil Hong; Son, Jaebum; Lim, Chae Seung; Kang, Jaewoo; Cha, Jihun; Park, Kijung; Kim, Dong Min | 2014 | Development of Test Toolkit of Hard Review to Evaluate a Random Clinical Decision Support System for the Management of Chronic Adult Diseases | Wireless Personal Communications | 79(4) | Not pre-discussed intervention |
| 32 | An, Ah Reum; Shin, Dong Wook; Chun, So Hyun; Lee, Hyun-Ki; Ko, Young-Jin; Lee, Hyejin; Son, Ki Young; Choi, Ho-Chun; Cho, Belong; Lee, Jong-koo; Kim, Jung Gu | 2014 | Shared decision-making on the use of hormone therapy: a nationwide survey in the Republic of Korea | Menopause | 21(7) | Not pre-discussed intervention |
| 33 | Lee, Eun Young | 2015 | Implications of Decision Coaching in Shared Decision Making | Korean Journal of Medical Ethics | 18(2) | Not pre-discussed designs |
| 34 | Seo, Minjeong | 2015 | 생애말기 암환자의 의료적 의사결정에 대한 질적 서술적 연구 | The Korean Academy of Adult Nursing Conference Proceedings | 2015 | Not pre-discussed intervention |
| 35 | Kim, Se Ik; Lee, Yumi; Son, Yedong; Jun, So Yeun; Yun, Sooin; Bae, Hyo Sook; Lim, Myong Cheol; Jung, So-Youn, Joo, Jungnam; Lee, Eun Sook | 2015 | Assessment of Breast Cancer Patients' Knowledge and Decisional Conflict Regarding Tamoxifen Use | Journal of Korean Medical Science | 30(11) | Not pre-discussed intervention |
| 36 | Park, Hayoung; Lee, Sang-il; Hwang, Hee; Kim, Yoon; Heo, Eun-Young; Kim, Jeong-Whun; Ha, Kyooseob | 2015 | Can a health information exchange save healthcare costs? Evidence from a pilot program in South Korea | International Journal of Medical Informatics | 84(9) | Not pre-discussed intervention |
| 37 | Yeun, Eun Ja; Kwon, Young Mi; Kim, Jung A | 2015 | Decision-making regarding organ donation in Korean adults: A grounded-theory study | Nursing & health sciences | 17(2) | Not pre-discussed outcomes |
| 38 | Jo, Kae-Hwa & An, Gyeong-Ju | 2015 | Effects of an educational programme on shared decision-making among Korean nurses | International Journal of Nursing Practice | 21(6) | Not pre-discussed participants |
| 39 | Jo, Kae-Hwa; An, Gyeong-Ju; Lee, Hong Seon Lee | 2015 | Health Care Professional Factors Influencing Shared Medical Decision Making in Korea | Sage Open | 5(4) | Not pre-discussed intervention |
| 40 | Song, Yoon Jin | 2016 | Limitations and alternatives of autonomy competence model in medical decision making | Korean journal of legal philosophy | 19(3) | Not pre-discussed intervention |
| 41 | Lee, Na-Yeon; Lee, Seungjin; Lee, Soo-Kyoung | 2016 | Perceptions and Needs of Hospice Palliative Care and Shared Decision Making among Middle-Aged Adults | The Korean journal of hospice and palliative care | 19(4) | Not pre-discussed participants |
| 42 | Yi, Myungsun; Joung, Woo Joung; Park, Eun Young; Kwon, Eun Jin; Kim, Haejin; Seo, Ji Young | 2016 | Decision Making Experience on Breast Reconstruction for Women with Breast Cancer. [Korean] | Journal of Korean Academy of Nursing | 46(6) | Not pre-discussed designs |
| 43 | Shin, Dong Wook; Cho, Juhee; Debra L. Roter; Kim, So Young; Yang, Hyung Kook; Park, Keeho; Kim, Hyung Jin; Shin, Hee-Young; Kwon, Tae Gyun; Park, Jong Hyock | 2017 | Attitudes Toward Family Involvement in Cancer Treatment Decision Making: The Perspectives of Patients, Family Caregivers, and Their Oncologists | Psycho-oncology | 26(6) | Not pre-discussed intervention |
| 44 | Kim, Kkotbong & Yang, Jinhyang | 2017 | Decision-making process related to treatment and management in Korean women with breast cancer: Finding the right individualized healthcare trajectory | Applied nursing research: ANR | 35 | Not pre-discussed designs |
| 45 | Andrew P. Brogan; Carla DeMuro; Amy M. Barrett; Denise D’Alessio; Vasudha Bal; Susan L. Hogue | 2017 | Payer perspectives on patient-reported outcomes in health care decision making: Oncology examples | Journal of Managed Care and Specialty Pharmacy | 23(2) | Not pre-discussed designs |
| 46 | Sim, Jin Ah; Chang, Yoon Jung; Shin, Aesun; Noh, Dong-Young; Han, Wonshik; Yang, Han-Kwang; Kim, Young Whan; Kim, Young Tae; Jeong, Seoung-Yong; Yoon, Jung-Hwan; Kim, Yoon Jun; Heo, Daesuk; Kim, Tae-You; Oh, Do-Youn; Wu, Hong-Gyun; Kim, Hak Jae; Chie, Eui Kyu; Kang, Keon Wook; Kim, Ju Han; Yun, Young | 2017 | Perceived needs for the information communication technology (ICT)-based personalized health management program, and its association with information provision, health-related quality of life (HRQOL), and decisional conflict in cancer patients | Psycho-Oncology | 26(11) | Not pre-discussed intervention |
| 47 | Cha, Jae Myung; Park, Dong Il; Park, Sang Hyoung; Shin, Jeong Eun; Kim, Wan Soo; Yang, Suk-Kyun | 2017 | Physicians Should Provide Shared Decision-Making for Anti-TNF Therapy to Inflammatory Bowel Disease Patients | Journal of Korean medical science | 32(1) | Not pre-discussed designs |
| 48 | Bae, Jong-Myon | 2017 | Strategies for Appropriate Patient-centered Care to Decrease the Nationwide Cost of Cancers in Korea | Journal of Preventive Medicine and Public Health | 50(4) | Not pre-discussed designs |
| 49 | Lee, Suk-Hyang; Hong, Juhee; 염지혜 | 2018 | A Qualitative Inquiry on Medical Service Improvement and Support Needs based on Medical Services and Self-Determination Experiences of People with Developmental Disabilities | Korean Journal of Physical, Multiple, & Health Disabilities | 61(2) | Not pre-discussed participants |
| 50 | 이현주; 오의금; 김수; 김상희; 김용찬; 이윤진 | 2018 | Shared decision making of early diagnosed cancer patients | Asian Nursing Research Conference Proceedings | 2018 | Not pre-discussed intervention |
| 51 | Kim, Yun Jae & Song, Jun Ah | 2018 | Perception about Shared Decision Making of Family Caregivers of Early Dementia Patients: A Qualitative Content Analysis Study | Journal of the Korean Gerontological Society | 38(3) | Not pre-discussed intervention |
| 52 | Yoon, Nan-He | 2018 | Patients' Participation in Treatment Decision Making and Health Status | Quality improvement in health care | 24(1) | Not pre-discussed outcomes |
| 53 | Kim, Kyounghae; Qian-Li Xue; Benita Walton-Moss; Marie T. Nolan; Han, Hae-Ra | 2018 | Decisional balance and self-efficacy mediate the association among provider advice, health literacy and cervical cancer screening | European Journal of Oncology Nursing | 32 | Not pre-discussed intervention |
| 54 | Lee, Hye Ran Lee; Lim, Chiyeon; Yun, Hyong Geun; Kang, Seung Hee; Kim, Do Yeun | 2018 | Making an informed decision of Korean cancer patients: the discrepancy between a patient's recall of information and the information needed for acquisition of radiotherapy informed consent | Supportive Care in Cancer | 26(1) | Not pre-discussed intervention |
| 55 | Juan P. Brito; Moom, Jae Hoon; Rebecca Zeuren; Kong, Sung Hye; Kim, Yeo Goon; Nicole M. Iñiguez-Ariza; Choi, June Young; Lee, Kyu Eun; Kim, Ji-hoon; Ian Hargraves; Victor Bernet; Victor M. Montori; Park, Young Joo; R. Michael Tuttle | 2018 | Thyroid Cancer Treatment Choice: A Pilot Study of a Tool to Facilitate Conversations with Patients with Papillary Microcarcinomas Considering Treatment Options | Thyroid | 28(10) | Not pre-discussed participants |
| 56 | Lee, Won-Suk; Ahn, Sung Min; Chung, Jun-Won; Kim, Kyoung Oh; Kwon, Kwang An; Kim, Yoonjae; Sym, Sunjin; Shin, Dongbok, Park, Inkeun; Lee, Uhn; Baek, Jeong-Heum | 2018 | Assessing Concordance With Watson for Oncology, a Cognitive Computing Decision Support System for Colon Cancer Treatment in Korea | JCO Clinical Cancer Informatics | 2 | Not pre-discussed outcomes |
| 57 | Choi, Im-Soon; Choi, Eun Young; Lee, Iyn-Hyang | 2019 | Challenges in informed consent decision-making in Korean clinical research: A participant perspective | Plos One | 14(5) | Not pre-discussed outcomes |
| 58 | choi, Youn I; Chung, Jun-won; Kim, Kyoung Oh; Kwon, Kwang An; Kim, Yoon Jae; Park, Dong Kyun; Ahn, Sung Min; Park, So Hyun; Sym, Sun jin; Shin, Dong Bok; Kim, Young Saing; Sung, Ki Hoon; Baek, Jeong-Heum; Lee, Uhn | 2019 | Concordance Rate between Clinicians and Watson for Oncology among Patients with Advanced Gastric Cancer: Early, Real-World Experience in Korea | Canadian Journal of Gastroenterology and Hepatology | 2019 | Not pre-discussed participants |
| 59 | K. H. Yoo; Y. A. Zhang; E. K. Yun | 2019 | Registered Nurses (RNs)' knowledge sharing and decision-making: the mediating role of organizational trust | International Nursing Review | 66(2) | Not pre-discussed participants |
| 60 | Kim, Eui Joo; Woo, Hyun Sun; Cho, Jae Hee; Sym, Sun Jin; Baek, Jeong-Heum; Lee, Won-Suk; Kwon, Kwang An; Kim, Kyoung Oh; Chung, Jun-Won; Park, Dong Kyun; Kim, Yoon Jae | 2019 | Early experience with Watson for oncology in Korean patients with colorectal cancer | PLoS One | 14(3) | Not pre-discussed outcomes |
| 61 | Kim, Su-young & Lee, Jihae | 2020 | A study of participation in health-related decision and self-care competency in patients with chronic disease | Journal of Korea Academia-Industrial cooperation Society | 21(6) | Not pre-discussed designs |
| 62 | Ko, Eunkyong & Yeo, Jungsung | 2020 | Rights of Healthcare Consumers and Shared Decision-making in Medical Services | Journal of Consumer Studies | 31(2) | Not pre-discussed outcomes |
| 63 | Kim, Joo-Young; Lee, Kyoung Eun; Kim, Kyubo; Lee, Myung Ah; Yoon, Won Sup; Han, Dong Seok; Ahn, Sung Gwe; Kang, Jung-Hun | 2020 | Choosing Wisely: The Korean Perspective and Launch of the 'Right Decision in Cancer Care' Initiative | Cancer research and treatment | 52(3) | Not pre-discussed participants |
| 64 | Kim, Jung Sun; Yoo, Shin Hye; Choi, Wonho; Kim, Yejin; Hong, Jinui; Kim, Min Sun; Park, Hye Yoon; Keam, Bhumsuk; Heo, Dae Seog | 2020 | Implication of the Life-Sustaining Treatment Decisions Act on End-of-Life Care for Korean Terminal Patients | Cancer Research and Treatment | 52(3) | Not pre-discussed intervention |
| 65 | Lee, Kyounga & Lee, Seon Heui | 2020 | Artificial Intelligence-Driven Oncology Clinical Decision Support System for Multidisciplinary Teams | Sensors (Basel) | 20(17) | Not pre-discussed designs |
| 66 | Lee, Jihae | 2021 | Experience of Patients' Having Hypertension and Diabetes Participation in Health Care | The Journal of the Korea Contents Association | 21(3) | Not pre-discussed participants |
| 67 | Jeong, Miri | 2021 | A Study on the Mediating Effect of Patient Activation between Trust in Healthcare Professionals and Shared Decision Making in Diabetic Patients | Journal of the Korea Convergence Society | 12(9) | Not pre-discussed outcomes |
| 68 | An, Bomi & Lee, Jihae | 2021 | A Study of Autonomy Preference on Decision-making among Patients with Chronic Disease | The Journal of the Korea Contents Association | 21(1) | Not pre-discussed outcomes |
| 69 | Kim, Soojin; An, Soontae; Kim, Sejoong; Ryu, Dong-Ryeol; Kim, Hwanhee | 2021 | An exploratory study on the shared decision-making as a two-way symmetrical communication process: Focusing on the communication of kidney dialysis patients* | Korean Journal of Journalism & Communication Studies | 65(2) | Not pre-discussed participants |
| 70 | Lee, Soyoung | 2021 | Strategien der Ärzte zur Selbstbestimmung der Patienten in der medizinischen Kommunikation - Hinsichtlich der Perspektivendivergenzen | Koreanische Zeitschrift fur deutschunterricht | 80(80) | Not pre-discussed intervention |
| 71 | Park, Hye Jung & Jang, In Sun | 2021 | Factors influencing self-care agency of chronic diseases with disabilities: Focused on health literacy, social support and perceived involvement in care | Journal of Korean Academy on Communication in Healthcare | 16(2) | Not pre-discussed outcomes |
| 72 | Kim, Hyeyeong; Im, Hyeon-Su; Lee, Kyong Og; Min, Young Joo; Jo, Jae-Cheol; Choi, Yunsuk; Lee, Yoo Jin; Kang, Daseul; Kim, Changyoung; Koh, Su-Jin; Cheon, Jaekyung | 2021 | Changes in decision-making process for life-sustaining treatment in patients with advanced cancer after the life-sustaining treatment decisions-making act | BMC Palliative Care | 20(1) | Not pre-discussed outcomes |
| 73 | Park, So-Youn; Lee, Bomyee; Seon, Jeong Yeon; Oh, In-Hwan | 2021 | A National Study of Life-Sustaining Treatments in South Korea: What Factors Affect Decision-Making? | Cancer Research and Treatment | 53(2) | Not pre-discussed intervention |
| 74 | Sohn, Kate J.; Park, Sun-Young; Kim Sue | 2021 | A scoping review of return to work decision-making and experiences of breast cancer survivors in Korea | Supportive Care in Cancer | 29(4) | Not pre-discussed designs |
| 75 | Choi, Jiyeon | 2022 | Integrative Review of the Components of Shared Decision-Making | Korean Journal of Medical Ethics | 25(1) | Not pre-discussed intervention |
| 76 | Chang, Yoon Jung; Cho, Seungyeon; Joo, Jungnam; Ryu, Kum Hei; Lee, Sangwon; Cho, Juhee; Lim, Myong Cheol; Jung, So-Youn; Han, Jai Hong; Lee, Eun Sook; Kong, Sun-Young | 2022 | Differences in Willingness to Undergo BRCA1/2 Testing and Risk Reducing Surgery among the General Public, Cancer Patients, and Healthcare Professionals: A Large Population-Based Survey | Journal of Personalized Medicine | 12(5) | Not pre-discussed designs |
| 77 | Yu, Byung Chul; Han, Miyeun; Ko, Gang-Jee; Yang, Jae Won; Kwon, Soon Hyo; Chung, Sungjin; Hong, Yu Ah; Hyun, Young Youl; Cho, Jang-Hee; Yoo, Kyung Don; Bae, Eunjin; Park, Woo Yeong; Sun, In O; Kim, Dongryul; Kim, Hyunsuk; Hwang, Won Min; Song, Sang Heon; Shin, Sung Joon | 2022 | Effect of shared decision-making education on physicians' perceptions and practices of end-of-life care in Korea | Kidney Research and Clinical Practice | 41(2) | Not pre-discussed outcomes |
| 78 | Ahn, Shinae | 2022 | The Effects of Outpatients’ Experiences of Patient Participation on Patient Satisfaction in Korea | The Journal of Korean Nursing Administration Academic Society | 28(2) | Not pre-discussed outcomes |
| 79 | Kim, Do Kyong & Kim, Wook-Joo | 2023 | Ethical Analysis of the Decision-making Process for Patients with Acute Severe Stroke | Korean Journal of Medical Ethics | 26(2) | Not pre-discussed outcomes |
| 80 | Kim, Jikyeong | 2023 | Comparison of informed consent and shared decision-making concepts | Journal of the Korea Bioethics Association | 24(1) | Not pre-discussed designs |
| 81 | Kim, Da Eun & Kim, Min Jung | 2023 | Factors influencing shared decision-making in long-term care facilities | BMC Geriatrics | 23(1) | Not pre-discussed intervention |
| 82 | Baek, Soo Yeon; Kim, Hong-Kyu; Park, Seho; Yu, Jong Han; Lee, Min Hyuk; Young, Hyun Jo; Kim, Hyun-Ah; Han, Jai Hong; Choi, Jung Eun; Lee, Jung Ryeol; Lee, Kyung-Hun; Chung, Seockhoon; Chae, Hee Dong; Kim, Seonok; Yoo, Soyoung; Hahm, Sang Keun; Kim, Hee Jeong | 2023 | Multidisciplinary Shared Decision Making for Fertility Preservation in Young Women With Breast Cancer | Journal of breast cancer | 26(6) | Not pre-discussed intervention |
| 83 | Kim, Jin Eop; Park, Woo Yeong; Kim, Hyunsuk | 2023 | Renal Replacement Therapy For Elderly Patients with ESKD Through Shared Decision-Making | Electrolyte and Blood Pressure | 21(1) | Not pre-discussed intervention |
| 84 | Chang, Jae Hyun; Kim, Yong Chul; Song, Sang Heon; Kim, Soojin; Jo, Min-Woo; Kim, Sejoong | 2023 | Shared Decision Making for Choosing Renal Replacement Therapy in Chronic Kidney Disease Patients (SDM-ART trial): study protocol for randomized clinical trial | Kidney research and clinical practice | 42(6) | Not pre-discussed intervention |
| 85 | Lee, Sang-Hoon; Park, YoungJu; Choi, Chan-Bum; Kim, Yong-Gil; Kim, Jung-Ae; Cha, Hoon-Suk | 2023 | Usability and understandability of a web-based medical communication aid for patients with ankylosing spondylitis in South Korea: A mixed-methods study | Medicine (Baltimore) | 102(14) | Not pre-discussed designs |
| 86 | Park, Jinkyung; Lee, Joochul; Kim, Kyounghae | 2023 | Pathways Through Which Experience in Treatment Decision-Making Affects Health-Related Quality of Life in People with Chronic Conditions | Journal of Health Communication | 28(7) | Not pre-discussed designs |
| 87 | Chong, Hye Jin; Jang, Min Kyeong; Kim, Hyun Kyung | 2024 | Decision-making experiences regarding kidney transplant among older adults in South Korea: A qualitative descriptive study | Patient Education and Counseling | 119 | Not pre-discussed intervention |
| 88 | Byun, Hwa Kyung; Koon, Woong Sub; Park, Se-Jun; Kim, Sang-Il; Kim, Jin Ho; Kim, Young-Hoon; Chang, Bong-Soon; Ahn, Yong Chan | 2024 | Different decision-making in spine metastasis management among radiation oncologists and orthopedic surgeons: a Korean online survey study | Frontiers in Neurology | 14 | Not pre-discussed intervention |
| 89 | Yun, Ji Young; Jeon, Dong Nyeok; Jeon, Byung-Joon; Kim, Eun Key | 2024 | Factors influencing the decision-making process in breast reconstruction from the perspective of reconstructive surgeons: A qualitative study involving Korean plastic surgeons | Journal of Plastic, Reconstructive & Aesthetic Surgery | 93 | Not pre-discussed intervention |
| 90 | Lee, Seonah | 2024 | Association Between Korean Adults' Electronic Health Literacy and Active Participation in Health Decision-Making | Computers, informatics, nursing | 7 | Not pre-discussed intervention |
| 91 | Kim, Ji-Jyeong | 2024 | Shared Decision Making in Geriatric Care | Korean Journal of Geriatrics & Gerontology | 25(1) | Not pre-discussed outcomes |

# Appendix D: Quality assessment

The criteria for evaluating the bias of the selected studies are as shown in the table below.

Supplement Table 9. Evaluation criteria agreed upon by researchers

| **Bias** | **Authors’ assessment**  **of risk of bias** | **Support for assessment** |
| --- | --- | --- |
| 1. Question or objective  sufficiently described? | Yes | Easily identifiable in the Introduction (or first paragraph of the Methods section) |
|  | Partial | Vague/incomplete; or some information must be gathered from parts of the paper other than the introduction/background/objectives section. |
|  | No | Question or objective is not reported, or is incomprehensible. |
| 2. Study design evident and appropriate? | Yes | The design is easily identifiable and appropriate to address the study question/objective. |
|  | Partial | The design and/or research question(s) are not clearly identified, but can be seen in parts of the paper. |
|  | No | The design used does not answer the study question; or the design cannot be identified. |
| 3. Method of subject/comparison group selection or source of information/input variables described and appropriate?. | Yes | Inclusion/exclusion criteria designed to obtain an unbiased sample of the relevant target population or the entire population of interest are described and defined. For volunteer studies, recruitment methods and settings should be reported. |
|  | Partial | Selection methods (and inclusion/exclusion criteria, where applicable) are not completely described, but no obvious inappropriateness. Or selection strategy is likely introduced bias, but did not likely seriously distort the results. Target population mentioned but sampling strategy unclear. |
|  | No | No information provided. Or presence of selection bias that is likely to have seriously biased the results |
| 4. Subject (and comparison group, if applicable) characteristics or input  variables/information sufficiently described? | Yes | Sufficient relevant baseline/demographic information is provided or defined to clearly characterize or categorize participants. Baseline estimates for input variables are clearly stated. |
|  | Partial | The criteria is poorly defined or the relevant baseline/demographic information is incomplete. Reporting incomplete baseline estimates for input variables. |
|  | No | No baseline / demographic information provided.  Baseline estimates of input variables not given. |
| 5. If random allocation to treatment group was possible, is it described? | Yes | True randomization is complete - the method used is described. |
|  | Partial | Randomization mentioned, but method is not. |
|  | No | Randomization was possible, but not enforced or mentioned. |
|  | N/A | A study that does not require a random sample or is not an RCT. |
| 6. If interventional and blinding of investigators was possible, was it reported? | Yes | Blinding reported. |
|  | Partial | Blinding reported but it is not clear who was blinded. |
|  | No | Blinding would have been possible (and was possibly done) but is not reported |
|  | N/A | Study that does not require blinding or is not an RCT. |
| 7. If interventional and blinding of subjects was possible, was it reported? | Yes | Blinding reported. |
|  | Partial | Blinding reported but it is not clear who was blinded |
|  | No | Blinding would have been possible (and was possibly done) but is not reported. |
|  | N/A | Study that does not require blinding or is not an RCT. |
| 8. Outcome and (if applicable) exposure measure(s) well defined and robust to measurement / misclassification bias? Means of assessment reported? | Yes | Exposure and outcome measures are defined and assessed using a tool with validated reliability or validity |
|  | Partial | Definitions of exposure and outcome leave room for subjective interpretation or are not reported in sufficient detail, but are verifiable, e.g., through results tables, and there is no evidence that the paper is likely to be materially compromised. |
|  | No | Measures of exposure and outcomes were undefined or inconsistent, measured using instruments that had not been validated for reliability or validity, and described without clearly presenting the results. |
| 9. Sample size appropriate? | Yes | A program such as G-power was used to calculate the appropriate sample size for the analysis, or a sample of at least 100 people was generally considered an appropriate sample size. |
|  | Partial | Insufficient data to assess sample size or differences in sample size between groups being compared. |
|  | No | If the sample size is too small (less than 30) or clearly inappropriate |
| 10. Analytic methods described and appropriate? | Yes | Analytical methods (e.g. chi-square, t-tests, etc.) are described and appropriate. |
|  | Partial | Methods of analysis not reported, but can be inferred from the results table. |
|  | No | The analytical methods are not described and cannot even be estimated. |
| 11. Some estimate of variance is reported for the main results? | Yes | Report appropriate estimates of variance (e.g., range, distribution, standard deviation, confidence intervals, etc.). |
|  | Partial | Variance estimates not provided for all key outcomes/results |
|  | No | No information about the variance estimate. |
| 12. Controlled for confounding? | Yes | Homogeneous subjects with no differences between groups at baseline, reported comparability, appropriate random sampling. |
|  | Partial | Incomplete control of confounders. Or control for confounders was reported but not fully explained. Or randomized trials without reporting comparability of baseline characteristics. Or confounders were not controlled for, but were unlikely to have significantly biased the results. |
|  | No | Confounding was not accounted for and may have seriously biased the results. |
| 13. Results reported in sufficient detail? | Yes | The results include all of the results mentioned. |
|  | Partial | If only partial results were reported instead of the full results, or if a table was provided but without a detailed description, or if no table was provided but was described in some technical terms |
|  | No | When the results of a subanalysis are presented without reporting the overall results, or when the results are not presented as values but are replaced by vague descriptions such as "more likely". |
| 14. Conclusions supported by the results? | Yes | When all conclusions are supported by the data. Conclusions are based on both negative and positive findings relevant to the research question. |
|  | Partial | Some conclusions are written in addition to, rather than in place of, the results, and extend beyond the results into the realm of interpretation of the data. Unnecessary links between results and conclusions. |
|  | No | Drawing conclusions from results that are not presented or explained, and drawing conclusions based on previous research rather than the results. |

# Appendix E: Preferred Reporting Items for Systematic reviews and Meta-Analyses extension for Scoping Reviews (PRISMA-ScR) Checklist

Supplementary table S 10. Preferred Reporting Items for Systematic reviews and Meta-Analyses extension for Scoping Reviews (PRISMA-ScR) Checklist

| **Section and Topic** | **Item #** | **Checklist item** | **Location where item is reported** |
| --- | --- | --- | --- |
| **TITLE** | | |  |
| Title | 1 | Identify the report as a systematic review. | p. 1 |
| **ABSTRACT** | | |  |
| Abstract | 2 | Provide a structured summary including, as applicable: background; objectives; data sources; study eligibility criteria, participants, and interventions; study appraisal and synthesis methods; results; limitations; conclusions and implications of key findings; systematic review registration number. | Written separately |
| **INTRODUCTION** | | |  |
| Rationale | 3 | Describe the rationale for the review in the context of existing knowledge. | p. 1 |
| Objectives | 4 | Provide an explicit statement of the objective(s) or question(s) the review addresses. | p. 1-2 |
| **METHODS** | | |  |
| Eligibility criteria | 5 | Specify the inclusion and exclusion criteria for the review and how studies were grouped for the syntheses. | p. 2-3  Appendix A |
| Information sources | 6 | Specify all databases, registers, websites, organisations, reference lists and other sources searched or consulted to identify studies. Specify the date when each source was last searched or consulted. | p. 3  Appendix B |
| Search strategy | 7 | Present the full search strategies for all databases, registers and websites, including any filters and limits used. | p. 3  Appendix B |
| Selection process | 8 | Specify the methods used to decide whether a study met the inclusion criteria of the review, including how many reviewers screened each record and each report retrieved, whether they worked independently, and if applicable, details of automation tools used in the process. | p. 3, Figure1 |
| Data collection process | 9 | Specify the methods used to collect data from reports, including how many reviewers collected data from each report, whether they worked independently, any processes for obtaining or confirming data from study investigators, and if applicable, details of automation tools used in the process. | p. 3-4 |
| Data items | 10a | List and define all outcomes for which data were sought. Specify whether all results that were compatible with each outcome domain in each study were sought (e.g. for all measures, time points, analyses), and if not, the methods used to decide which results to collect. | p. 4 |
|  | 10b | List and define all other variables for which data were sought (e.g. participant and intervention characteristics, funding sources). Describe any assumptions made about any missing or unclear information. | p. 4 |
| Study risk of bias assessment | 11 | Specify the methods used to assess risk of bias in the included studies, including details of the tool(s) used, how many reviewers assessed each study and whether they worked independently, and if applicable, details of automation tools used in the process. | p. 4  Appendix D |
| Effect measures | 12 | Specify for each outcome the effect measure(s) (e.g. risk ratio, mean difference) used in the synthesis or presentation of results. | NA |
| Synthesis methods | 13a | Describe the processes used to decide which studies were eligible for each synthesis (e.g. tabulating the study intervention characteristics and comparing against the planned groups for each synthesis (item #5)). | p. 3-4 |
|  | 13b | Describe any methods required to prepare the data for presentation or synthesis, such as handling of missing summary statistics, or data conversions. | NA |
|  | 13c | Describe any methods used to tabulate or visually display results of individual studies and syntheses. | p. 3-4 |
|  | 13d | Describe any methods used to synthesize results and provide a rationale for the choice(s). If meta-analysis was performed, describe the model(s), method(s) to identify the presence and extent of statistical heterogeneity, and software package(s) used. | p. 3-4 |
|  | 13e | Describe any methods used to explore possible causes of heterogeneity among study results (e.g. subgroup analysis, meta-regression). | NA |
|  | 13f | Describe any sensitivity analyses conducted to assess robustness of the synthesized results. | NA |
| Reporting bias assessment | 14 | Describe any methods used to assess risk of bias due to missing results in a synthesis (arising from reporting biases). | Appendix D |
| Certainty assessment | 15 | Describe any methods used to assess certainty (or confidence) in the body of evidence for an outcome. | NA |
| **RESULTS** | | |  |
| Study selection | 16a | Describe the results of the search and selection process, from the number of records identified in the search to the number of studies included in the review, ideally using a flow diagram. | p. 4.  Figure 1 |
|  | 16b | Cite studies that might appear to meet the inclusion criteria, but which were excluded, and explain why they were excluded. | Figure 1  Appendix C |
| Study characteristics | 17 | Cite each included study and present its characteristics. | p. 5, Table 1 |
| Risk of bias in studies | 18 | Present assessments of risk of bias for each included study. | p. 7.  Figure 2, Table 1 |
| Results of individual studies | 19 | For all outcomes, present, for each study: (a) summary statistics for each group (where appropriate) and (b) an effect estimate and its precision (e.g. confidence/credible interval), ideally using structured tables or plots. | p. 5-7.  Table 2. 3 |
| Results of syntheses | 20a | For each synthesis, briefly summarise the characteristics and risk of bias among contributing studies. | p. 7.  Figure 2, Table 1 |
|  | 20b | Present results of all statistical syntheses conducted. If meta-analysis was done, present for each the summary estimate and its precision (e.g. confidence/credible interval) and measures of statistical heterogeneity. If comparing groups, describe the direction of the effect. | p. 5-7.  Table 2. 3 |
|  | 20c | Present results of all investigations of possible causes of heterogeneity among study results. | NA |
|  | 20d | Present results of all sensitivity analyses conducted to assess the robustness of the synthesized results. | NA |
| Reporting biases | 21 | Present assessments of risk of bias due to missing results (arising from reporting biases) for each synthesis assessed. | NA |
| Certainty of evidence | 22 | Present assessments of certainty (or confidence) in the body of evidence for each outcome assessed. | NA |
| **DISCUSSION** | | |  |
| Discussion | 23a | Provide a general interpretation of the results in the context of other evidence. | p. 7-10 |
|  | 23b | Discuss any limitations of the evidence included in the review. | p. 10 |
|  | 23c | Discuss any limitations of the review processes used. | p. 10 |
|  | 23d | Discuss implications of the results for practice, policy, and future research. | p. 10-11 |
| **OTHER INFORMATION** | | |  |
| Registration and protocol | 24a | Provide registration information for the review, including register name and registration number, or state that the review was not registered. | p. 2 |
|  | 24b | Indicate where the review protocol can be accessed, or state that a protocol was not prepared. | NA |
|  | 24c | Describe and explain any amendments to information provided at registration or in the protocol. | NA |
| Support | 25 | Describe sources of financial or non-financial support for the review, and the role of the funders or sponsors in the review. | p. 12 |
| Competing interests | 26 | Declare any competing interests of review authors. | p. 12 |
| Availability of data, code and other materials | 27 | Report which of the following are publicly available and where they can be found: template data collection forms; data extracted from included studies; data used for all analyses; analytic code; any other materials used in the review. | Appendix |

*From:*  Page MJ, McKenzie JE, Bossuyt PM, Boutron I, Hoffmann TC, Mulrow CD, et al. The PRISMA 2020 statement: an updated guideline for reporting systematic reviews. BMJ 2021;372:n71. doi: 10.1136/bmj.n71

For more information, visit: <http://www.prisma-statement.org/>

# Appendix F: The PRISMA 2020 abstract checklist

Supplementary table S 7. The PRISMA 2020 abstract checklist

| **Section and Topic** | **Item#** | **Checklist item** | **Reported (Yes/No)** |
| --- | --- | --- | --- |
| **TITLE** | | |  |
| Title | 1 | Identify the report as a systematic review. | Yes |
| **BACKGROUND** | | |  |
| Objectives | 2 | Provide an explicit statement of the main objective(s) or question(s) the review addresses. | Yes |
| **METHODS** | | |  |
| Eligibility criteria | 3 | Specify the inclusion and exclusion criteria for the review. | Yes |
| Information sources | 4 | Specify the information sources (e.g. databases, registers) used to identify studies and the date when each was last searched. | Yes |
| Risk of bias | 5 | Specify the methods used to assess risk of bias in the included studies. | Yes |
| Synthesis of results | 6 | Specify the methods used to present and synthesise results. | No |
| **RESULTS** | | |  |
| Included studies | 7 | Give the total number of included studies and participants and summarise relevant characteristics of studies. | Yes |
| Synthesis of results | 8 | Present results for main outcomes, preferably indicating the number of included studies and participants for each. If meta-analysis was done, report the summary estimate and confidence/credible interval. If comparing groups, indicate the direction of the effect (i.e. which group is favoured). | Yes |
| **DISCUSSION** | | |  |
| Limitations of evidence | 9 | Provide a brief summary of the limitations of the evidence included in the review (e.g. study risk of bias, inconsistency and imprecision). | Yes |
| Interpretation | 10 | Provide a general interpretation of the results and important implications. | Yes |
| **OTHER** | | |  |
| Funding | 11 | Specify the primary source of funding for the review. | No |
| Registration | 12 | Provide the register name and registration number. | No |

*From:*  Page MJ, McKenzie JE, Bossuyt PM, Boutron I, Hoffmann TC, Mulrow CD, et al. The PRISMA 2020 statement: an updated guideline for reporting systematic reviews. BMJ 2021;372:n71. doi: 10.1136/bmj.n71 For more information, visit: http://www.prisma-stateme
